# Critical Evaluation of Spectral Resolution Enhancement Methods for Raman Hyperspectra

**DOI:** 10.1177/00037028211061174

**Published:** 2021-12-22

**Authors:** H. Georg Schulze, Shreyas Rangan, Martha Z. Vardaki, Michael W. Blades, Robin F. B. Turner, James M. Piret

**Affiliations:** 1 Monte do Tojal, Hortinhas, Terena (São Pedro), Portugal; 2 Michael Smith Laboratories, 8166 The University of British Columbia, Vancouver, BC, Canada; 3 School of Biomedical Engineering, 8166 University of British Columbia, Vancouver, BC, Canada; 4 Department of Medical Physics, School of Health Sciences, 69156 University of Ioannina, Ioannina, Greece; 5 Department of Chemistry, 8166 The University of British Columbia, Vancouver, BC, Canada; 6 Department of Electrical and Computer Engineering, 8166 The University of British Columbia, Vancouver, BC, Canada; 7 Department of Chemical and Biological Engineering, 8166 The University of British Columbia, Vancouver, BC, Canada

**Keywords:** Raman spectroscopy, resolution enhancement, blind deconvolution, over-deconvolution, node narrowing, pseudospectra, moving window multiple peak fitting, two-dimensional correlation spectroscopy, chemical contrast images, Fityk

## Abstract

Overlapping peaks in Raman spectra complicate the presentation, interpretation, and analyses of complex samples. This is particularly problematic for methods dependent on sparsity such as multivariate curve resolution and other spectral demixing as well as for two-dimensional correlation spectroscopy (2D-COS), multisource correlation analysis, and principal component analysis. Though software-based resolution enhancement methods can be used to counter such problems, their performances often differ, thereby rendering some more suitable than others for specific tasks. Furthermore, there is a need for automated methods to apply to large numbers of varied hyperspectral data sets containing multiple overlapping peaks, and thus methods ideally suitable for diverse tasks. To investigate these issues, we implemented three novel resolution enhancement methods based on pseudospectra, over-deconvolution, and peak fitting to evaluate them along with three extant methods: node narrowing, blind deconvolution, and the general-purpose peak fitting program Fityk. We first applied the methods to varied synthetic spectra, each consisting of nine overlapping Voigt profile peaks. Improved spectral resolution was evaluated based on several criteria including the separation of overlapping peaks and the preservation of true peak intensities in resolution-enhanced spectra. We then investigated the efficacy of these methods to improve the resolution of measured Raman spectra. High resolution spectra of glucose acquired with a narrow spectrometer slit were compared to ones using a wide slit that degraded the spectral resolution. We also determined the effects of the different resolution enhancement methods on 2D-COS and on chemical contrast image generation from mammalian cell spectra. We conclude with a discussion of the particular benefits, drawbacks, and potential of these methods. Our efforts provided insight into the need for effective resolution enhancement approaches, the feasibility of these methods for automation, the nature of the problems currently limiting their use, and in particular those aspects that need improvement.

## Graphical abstract

**Graphical Abstract:**
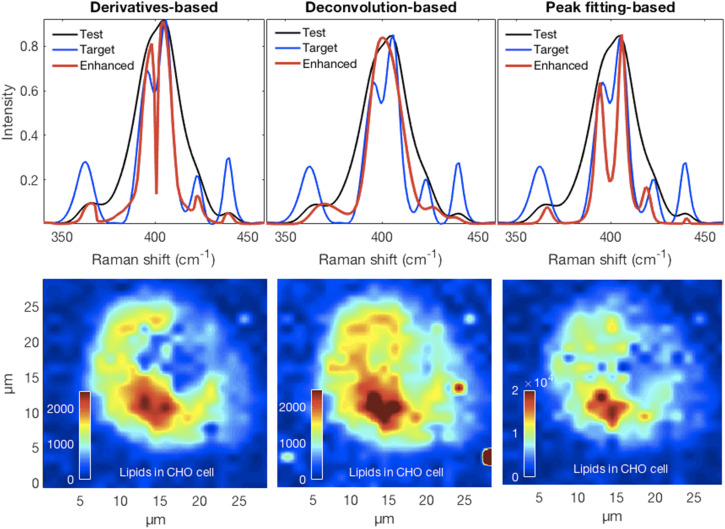

## Introduction

Raman bands consist of scattering intensities as a function of wavelength shift due to Raman scattering from polarizable molecular bonds,^1^ the ground truth, which are superimposed to produce an intrinsic Raman spectrum represented as the vector **s**. Even ground truth Raman bands can overlap. Overlap complicates the analysis of Raman spectra because it can obscure the number of bands as well as their parameter values, such as amplitudes, positions, widths, and shapes, which contain information about the scattering bonds and the chemical moieties present. Furthermore, intrinsic Raman spectra are measured with a spectrometer, a process that introduces distortions. Thus, the measured spectrum, represented by the vector **m**, is blurred by the measuring instrument point spread function (IPSF) that augments both the overlap of Raman bands and the peak parameter distortions. Given additional measurement noise, represented by the vector **n**, these relationships can be expressed as

(1) m=s∗ipsf+n

where ∗ denotes the convolution operator and **ipsf** is the IPSF in vector form. For scanned spectra, IPSFs tend to be Gaussian when predominantly affected by optical elements or triangular when dominated by slit effects.^2,3^ Due to these effects, for complex mixtures of different types of molecules, apportioning Raman bands to the correct originating molecular type and determining the correct band parameter values can be difficult.

The Raman spectra of biological cell and tissue samples consist of the superposition of scattering from numerous different macromolecular components. To identify the individual components and their relative concentrations, there is a need to “demix” such superpositions. Though Raman marker bands, unique to a given component, can be useful for identification and quantification purposes,^4^ this is often not possible when substantial band overlapping occurs. Nevertheless, a data search for those features that are linearly the most dissimilar to each other often becomes necessary because reduced complexity is desirable to implement many approaches such as multivariate curve resolution (MCR).^5^ Principal component analysis (PCA)^6,7^ and two-dimensional correlation spectroscopy (2D-COS) and its variants^8–12^ are dependent on the analysis of correlated changes between variables. However, where one band strongly diminishes in intensity, and has an overlapped and weaker neighbor with a modestly increasing intensity, the weaker band can exhibit an erroneously decreasing intensity. In general, independently changing overlapping bands can give rise to complex spectral features that are difficult to interpret. Accurate intensities, higher contrast ratios, and better spatial resolution obtainable with well-resolved bands would also benefit chemical contrast images based on Raman peaks,^13^ Raman PCA loadings,^14^ or individual demixed components. However, high resolution spectra are not strictly necessary for cell and tissue classification.^15^ The primary benefit of low resolution Raman spectroscopy appears to reside in overcoming detector noise leading to improved signal-to-noise ratios when using lower-grade, uncooled detectors.^16^ Low resolution Raman spectroscopy is also not suitable for cellular biochemical characterization and might not be effective for the classification of closely related cell types or different activation states of the same cell type. All the above considerations make resolution enhancement methods of interest to facilitate the study of complex samples.

Though instrumental methods exist to improve the resolution of collected spectra, for example, using narrow band lasers, narrow spectrometer entrance slits, high-resolution gratings, and multiphoton excitation,^3,17,18^ instrumental factors are often fixed, expensive, difficult to modify, or complicated to implement. Thus, computational methods can be by far the most feasible alternatives for resolution enhancement. But it must be understood that algorithmic methods are dependent on information captured during the measurement process, the use of constraints, and the availability of a priori information and this must limit one’s expectations of what can be achieved with them.

Resolution enhancement methods can generally be divided into three categories. These are methods of (i) band narrowing, (ii) deconvolution, and (iii) peak fitting. Narrowing methods are based on derivatives and power laws and have frequently been used to resolve overlapping peaks and improve image resolution.^19–24^ Second and fourth derivatives are especially useful because they produce peaks narrower than the originals, thus facilitating the identification of peaks.^25^ A derivative spectrum can yield a sensitive, qualitative profile for the characterization of compounds because subtle spectral features are emphasized that might otherwise be unobservable.^26^ Drawbacks include the rapid noise intensity increase upon differentiation^27^ and the appearance of satellite bands.^26^

Diverse methods have been developed to deconvolve the IPSF from the measured Raman bands, thus to computationally undo the effects of the IPSF.^2,3,28–33^ These have been characterized in three categories: non-blind deconvolution, semi-blind deconvolution and blind deconvolution.^34^ With non-blind deconvolution the IPSF is accurately known and deconvolution is frequently effected via Fourier transformation and division in the frequency domain.^35^ Fourier self-deconvolution is a related process where, instead of the IPSF, division occurs by a narrower band of the same shape as those in the spectra,^35,36^ leading to improved spectral resolution.^37^ With semi-blind deconvolution, the IPSF is not accurately known, but some information about it is known and optimization with regularization procedures are used for deconvolution.^38^ Blind deconvolution is used when the IPSF is not known and algorithms, such as the Richardson–Lucy algorithm,^39^ are used to obtain an estimate of both the IPSF and the IPSF-free spectrum.^34,39–43^ More recently, machine learning has added a fourth “implied deconvolution” method that makes no direct use of the IPSF. Instead, spectra are implicitly deconvolved by training algorithms to produce either deconvolved or super deconvolved spectra.^44–46^

The peak parameters of experimentally measured Raman bands are determined by fitting theoretical model profiles to these bands.^35,47^ For accurate fits, the most appropriate fitting profile should be selected given the nature of the Raman bands.^47^ Fitting can be applied to overlapping peaks, thus effectively deconvolving them.^35^ It has been used, for example, to identify, separate, and quantify the highly overlapping peaks in the amide I region of Raman protein spectra.^48^

The objective in this work was (i) to assess resolution enhancement of complex biological and biomedical Raman spectra by alternative methods, and so to facilitate their interpretation and presentation and (ii) to assess their ability to recover ground truth relative peak parameters for use by correlation-based analyses. These assessments were not intended to affect a head-to-head comparison between these methods, but to gain a deeper understanding of their performance characteristics in this context, seeking to reveal approaches that have particular promise for further adaptation and specific difficulties that need to be addressed. To this end, we investigated three existing methods, node narrowing (NN),^9^ blind deconvolution (BD) and the Fityk^49^ general purpose peak fitting program (FF) as well as three previously unpublished implementations based on envelope-shaped pseudospectra (EP), weighted over-deconvolution (WO) and moving window multiple peak fitting (MF). Of secondary concern was the potential for automation of these methods to cope with large biological and biomedical hyperspectral data sets that contain thousands or tens of thousands of spectra. Such large numbers of spectra cannot realistically be processed interactively, and fewer adjustable parameters tend to facilitate automation.^50,51^ In particular, given the increasing number of biomedical and therapeutic manufacturing applications of Raman spectroscopy,^52,53^ the quantitative interpretation of changes in the analyte should be performed reliably by automated methods.

We first introduce the methods and their implementation. Then, using a synthetic data set specifically crafted for this purpose, we determine the effects of different parameter settings on the method performances. From these results, guided by the estimated best parameter settings, we assess performance on resolution enhancement and the ability to recover the inherent correlation structure of the synthetic data set (i.e., the relationships of the correlations between the various peaks). We then apply these methods to real spectra of glucose obtained with a wide slit to degrade the spectral resolution and compare the results to high resolution spectra obtained with a narrow slit. Finally, we assess the methods using actual biological spectra obtained from mammalian cells. We then discuss their advantages and drawbacks and end with a conclusion based on the results.

## Methods

We attempted to enhance spectrometric resolution and effect ground truth recovery based on two spectral sharpening methods, NN and EP; two deconvolution methods, BD and WO; and two peak fitting methods, moving window multi-peak fitting and a fit-and-subtract fitting method. We briefly summarize each of these approaches below.

### Node Narrowing

Node narrowing^9^ is obtained with a filter of coefficients derived from a combination of first and second derivatives of a spectrum **a**_p_ to be filtered

(2) $$f\left(\text{ν}\right)=\;\exp\left[-\alpha \left\{1+\frac{a_{\text{p}}^{\prime \prime }\left(\text{ν}\right)a_{\text{p}}\left(\text{ν}\right)-\text{ε}}{\lambda {\left(a_{\text{p}}^{\prime }\left(\text{ν}\right)\right)}^{2}+\left|a_{\text{p}}^{\prime \prime }\left(\text{ν}\right)a_{\text{p}}\left(\text{ν}\right)\right|+\varepsilon }\right\}\right]$$

The filtered spectrum is given by

(3) $${\overset{ˇ}{a}}_{\text{p}}=f\left(\text{ν}\right)\cdot a_{\text{p}}$$

The two adjustable parameters, α and λ, were investigated for their effects on resolution enhancement; ε is present as a small value for computational stability.

### Envelope-Shaped Pseudospectra

A pseudospectrum^54^ is generated from the first derivative of a “parent” spectrum with the absolute values of the negative features shifted to coincide maximally with the positive features, summed with the positive features, and the sum smoothed using a moving average filter with a window size equal to the spectral resolution. The pseudospectrum is then scaled to the same area as the parent spectrum and shifted to maximize the correlation between it and the parent spectrum. The procedure is repeated, thus effectively relying on an approximation of a second derivative. Thereafter, an envelope shaping algorithm is used to scale the repeated pseudospectrum peaks to the intensities in the initial parent spectrum at their corresponding positions. This method has four adjustable parameters: an alignment setting to adjust the pseudospectra for envelope shaping, a setting for the spectral resolution, a setting for the degree of narrowing desired (approximating the derivative to use) and a smoothing order setting for the Savitzky–Golay smoothing when generating pseudospectra. We fixed the degree of narrowing at two and the smoothing order at zero to affect a reasonable tradeoff between peak recovery and noise, and then varied the other two adjustable parameters.

### Blind Deconvolution

The Matlab (The MathWorks lnc., USA) function, “deconvblind”, implements the maximum likelihood BD algorithm. It does not require a complete knowledge of the IPSF blurring but produces this as an output along with the deconvolved input. However, the number of elements of the IPSF vector strongly affects the results. Two parameters for this method were varied; the IPSF size and the noise suppressing damping argument level. The number of iterations were fixed at ten.

### Weighted Over-Deconvolution

We developed an algorithm using the Matlab “fmincon” optimization routine to deconvolve spectra with progressively larger IPSFs to narrow the peaks. The spectrum to be processed was also used to provide starting values for the procedure. These were obtained by shaping the squared values of the input peaks to their corresponding input values, thus providing narrow starting values. A weighting scheme was used to suppress satellite bands. The weights consisted of the input spectrum normalized to its maximum value. The deconvolved spectrum was then again convolved with the IPSF to approximate the original spectrum. The two-norm of the difference between the original spectrum and the reconvolution, element-wise multiplied with the weighting term, was used as the cost function to minimize.

Explanatory lines of code can be found in the Supplemental Material. This method had only two parameters to vary. Like the BD, there was one for the IPSF size and the other a damping threshold to further suppress artefacts.

### Moving Window Peak Fitting

A novel algorithm that we developed to fit distributions that could be Gaussian, Lorentzian, or Voigt-like was based on the Matlab “fit” function. Voigt peaks are peaks with mixed Lorentzian and Gaussian character and are often approximated with the Pearson VII distribution because the relative Lorentzian and Gaussian characters can be easily adjusted by varying a parameter.^2,35,50^ The method was devised to implement the simultaneous fitting of seven peaks in a moving window manner to process spectra containing more than seven peaks. The first four peaks’ parameters were recorded after the first fit converged. Thereafter, the first peak was dropped from the window and the next one added, then the next fit was performed. Only the parameters of the center peak were recorded. The procedure was repeated until the leading window edge reached the end of the spectrum at which point the last fit was performed and the parameters of all the remaining peaks recorded.

Starting values for peak heights and positions were obtained with a Matlab peak-finding algorithm (“findpeaks”) applied to envelope shaped once-repeated pseudospectra (i.e., second derivative-like). Starting widths were half the user-supplied spectral resolution of the input. Given a desired peak width, say 33% of the values recorded above, a new spectrum was generated from peak parameters with their widths artificially reduced to 33% of the original with commensurate increases in their peak heights.

The settings investigated pertained to peak positions and widths. Peak positions were constrained to vary from zero to five channels on either side of their starting positions. Peak widths were constrained to vary from one channel to three times the spectral resolution. Peak amplitudes were not constrained, but peaks less than 0.001 units were eliminated. Peak types were allowed to vary smoothly between fully Lorentzian or fully Gaussian.

### Fityk Peak Fitting

We deployed Fityk v.1.3.1 using the Lua scripting language^55^ using a Matlab shell. After loading a spectrum, initial peak Pearson VII (“Pearson7”) parameters for the tallest peak, except for the peak half-width at half-maximum (HWHM), were guessed provided that the tallest peak in the spectrum (or in the residual spectrum) exceeded the supplied minimum peak height threshold. A fit was then performed, the fitted peak subtracted from the original spectrum, and the procedure was repeated on the residual spectrum. This essentially implemented a fit-and-subtract approach. As above for the MF method, a new spectrum was generated from peak parameters with their widths artificially reduced to the given desired peak width of the original with commensurate increases in their peak heights.

The settings investigated were minimum height thresholds and peak widths. Peak height thresholds were varied from 0.01 to 0.049 in increments of 0.001 units (for peak intensities, see below). Initial peak widths were varied from 3 to 26 channels with one-channel increments.

### Synthetic Spectra

We first generated a 1000-channel vector with seven Lorentzian peaks that increasingly overlapped and two additional separately overlapping peaks for a total of nine peaks at channels 340, 465, 532, 584, 618, 645, 656, 800, and 813. The peak at channel 340 had an HWHM of six channels; for all other peaks, the HWHM was three channels. To obtain spectra with varied peak heights, every peak’s height was adjusted in successive steps as described below to generate ten synthetic spectra. The contribution from Peak 1 (P1, at 340) started from 0.15 and increased by 50%, at each change, up to 5.77. P2 started from 0.10 and increased linearly by 0.10 at each change. P3 decreased linearly from 1.00 to 0.95. P4 remained constant at 0.50 until step five, then from step six onward it increased linearly to 1.00. P5 was changed to be identical to P1. P6 decreased linearly from 1.00 to 0. P6 was highly overlapped with P7, such that P6 and P7 merged into one peak without a shoulder. Because P7 remained constant, this made it easy to determine any distortion of its peak height by the decreasing P6. The separately overlapping P8 and P9 remained constant. They provided a simple means to determine how consistently peak heights were recovered by each method and what distortions, if any, they caused.

**Figure 1.**
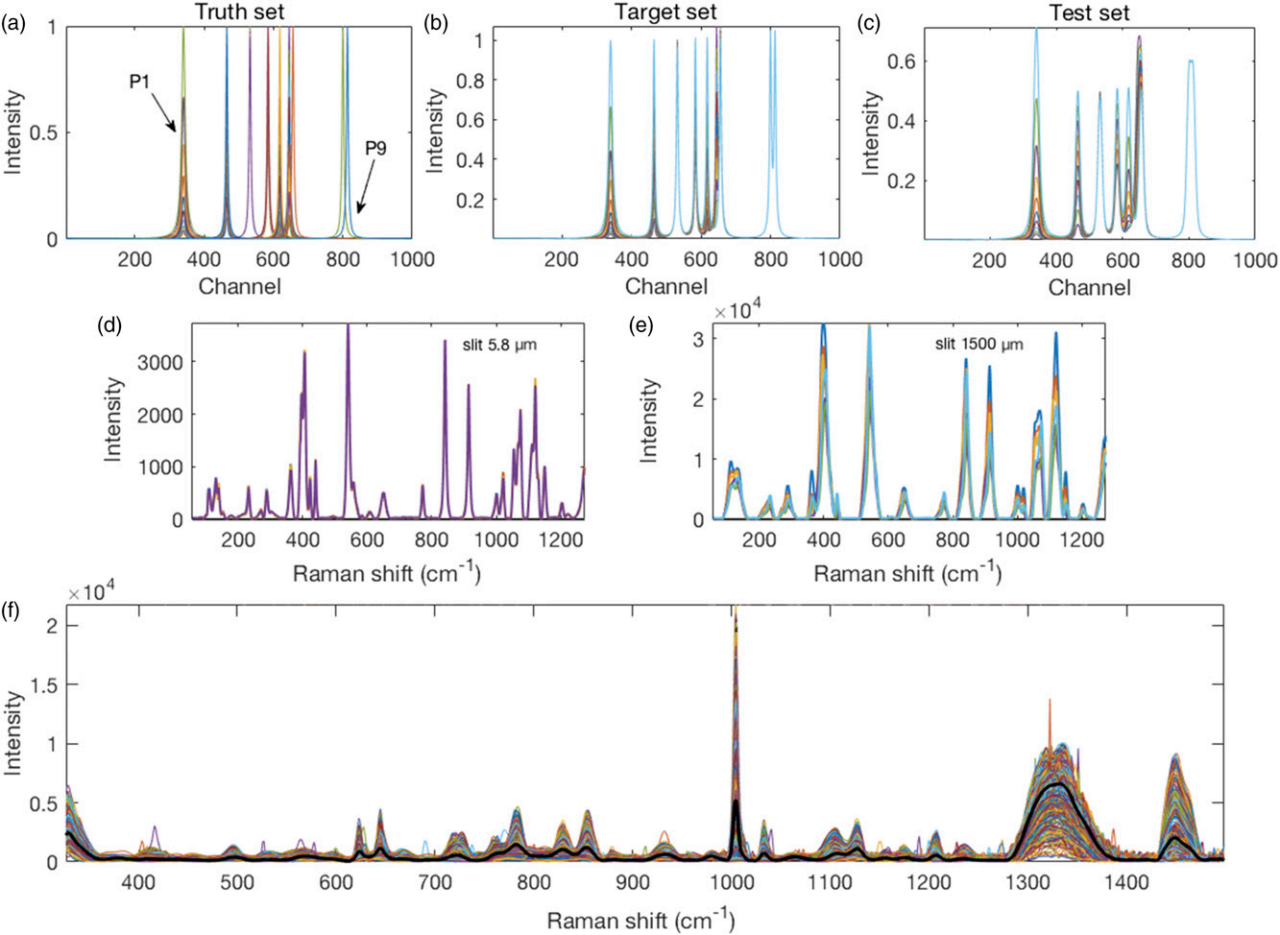
Synthetic spectra with nine peaks numbered consecutively from left (P1) to right (P9). (a) Individual Lorentzian peaks constituting the ground truth set were (b) assembled into 10 target set spectra. These were (c) convolved with a Gaussian distribution to create 10 test set spectra used for resolution enhancement. Real spectra from powdered glucose. (d) Spectra collected with a 5.8 µm slit width were used as the target set and (e) those collected with a 1500 µm wide slit were used as the test set. Due to the wider slit, these peaks were broader and some of them overlapped to cause adjacent peak fusion. For example, the grouping of five peaks centered around 400 cm^−1^ in (d) present now as only three peaks. (f) Colored traces of 462 preprocessed spectra raster scanned across a single mammalian CHO cell with their mean spectrum superimposed in black.

Within a set of changes for a given peak, the set was divided by the maximum value in that set to give a maximum value of 1.00 for the set (affected only P1 and P5). This set of peaks constituted the ground truth. A target spectrum was produced by combining all peaks of the ground truth set at a given stage to form a spectrum. Thereafter, the peaks in every set were convolved with an IPSF using a normal distribution with zero mean and a five-channel standard deviation. All convolved peaks at a given stage were then combined into a test spectrum. Thus, were generated ten synthetic target and ten synthetic test spectra, shown in Figs. 1a–c.

### Measured Raman Spectra

Spectra were collected from D-glucose powder (Invitrogen) with a confocal Raman microscope (lnVia, Renishaw, UK) using the operating software (WiRe), a 5× objective lens, and excitation with a 785 nm diode laser giving ∼150 mW power (∼3 Wmm^−2^) at the sample. For calibration, an external crystalline silicon standard was used. Different slit widths, 1500 µm and 5.8 µm, were used to manipulate the resolution of the spectra. One hundred glucose spectra obtained with 5 s integration time and a slit width of 1500 µm were averaged to provide a test spectrum. One hundred glucose spectra collected for 50 s each, with the slit open to 5.8 µm, were averaged to generate a target against which to evaluate the resolution enhancement methods applied to the test spectrum. Figures 1d and e shows the 5.8 µm and 1500 µm slit width spectra, respectively.

Chinese hamster ovary (CHO) cells are used for most recombinant protein production by the biotechnology industry. Chinese hamster Ovary cells were cultured by seeding 2.5x10^5^ CHO cells in shake flasks with 20 mL of chemically defined- (CD-) CHO medium (Gibco, USA), supplemented with 25 ng/mL recombinant human insulin-like growth factor-1 (hIGF-1), 4× anti-clumping agent, and 4 mM glutamine. Flasks were incubated in a humidified shaking incubator at 37 °C, 140 rpm, and 5% CO_2_ for 72 h. Exponentially growing cells were recovered from the flask, washed, and resuspended in a 15.4 mM NaCl solution in distilled water (0.09% saline). The resulting cell suspension was allowed to air dry on gold mirrors (Cat. No. PF05-03-M01, Thorlabs, USA) to obtain dry-fixed CHO cells. Raman spectroscopy was performed as above on a single cell with 40 s integration time, a 50× objective, a 50 µm slit width, and in map acquisition mode with the spectrometer centered at 950 cm^−1^.

### Data Generation and Processing

Matlab R2017b was used to simulate spectra, for spectral processing, and data analyses, except for FF as described above. A moving average-based baseline-flattening automated method^56^ and a two-dimensional second difference cosmic ray-induced spike removal method^57^ were used to preprocess measured spectra prior to smoothing with a fitting procedure.^58^ Computations were performed on a MacBook Air with a 2.2 GHz i7 processor and 8 GB 1600 MHz DDR3 memory running OS X Yosemite 10.10.5. More information about specific Matlab algorithms deployed here can be obtained from the Matlab help files.

### Evaluation Metrics

Spectral resolution enhancement needs to maintain relative peak heights, widths, positions, and shapes as these are of potential interest in a given application.^59^ It is furthermore important to avoid the introduction of artifacts.^19^ We evaluated improved spectral resolution based on the criteria below. These metrics were applied to the synthetic spectra, which could be best interpreted quantitatively by these methods. For the real spectra, we assessed resolution enhancement qualitatively or semi-quantitatively.

(i) To guard against processing-based spectral distortions, we recorded the root mean square error (RMSE) between the target and resolved sets of spectra

(4) rmseAll=RMSE(resolvedData, targetData)

We excluded the featureless parts below channel 200 and above channel 900.

(ii) We assessed the separation of two overlapping peaks, applied here to P8 and P9, as the height of the resolved spectrum’s smallest flanking peak minus the height of the valley between the overlapping peaks relative to the height of the same peak in the target spectrum minus the height of the corresponding valley in the target spectrum

(5) $$sep2=\frac{smallest\;resolved\;flanking\;peak-resolvedValley}{smallest\;target\;flanking\;peak-targetValley}$$

(iii) The reduction in peak HWHM was determined for freestanding peaks as the HWHM in the resolved spectrum relative to the corresponding HWHM in the target spectrum

(6) $$redW=\frac{resolvedPeakHWHM}{targetPeakHWHM}$$

A ten-step interpolation was used to obtain a more accurate HWHM for the resolved peaks, after processing with the resolution enhancement algorithms. We used P1 for these calculations.

Where quantitative results are desired or correlation dependent analyses need to be performed, the preservation of true peak positions and intensities in resolved spectra are required. Thus, we included in the evaluation (iv) the number of peaks in the first resolved spectrum of the series relative to those in the corresponding truth set, (v) the number of peaks in the resolved spectrum with incorrect positions relative to the truth set, and (vi) the mean deviation of peaks in the resolved spectrum from their correct positions.

Finally, we determined the correlations of the P6 and P7 intensities between the resolved spectra and the truth set because they were the most affected by overlap. If P6 and P7 were resolved, their intensities at the positions of their maxima were used, otherwise the positions used were those of the P6 and P7 maxima in the corresponding truth set. For increasingly well-resolved spectra, the correlation coefficient between P6 of the resolved set and P6 of the truth set (vii) should trend higher and ideally reach unity. In contrast, for increasingly well-resolved spectra, this correlation coefficient for P6 of the resolved set and P7 of the truth set (viii) should trend lower due to diminished overlap. However, as the truth set P7 was constant, a correlation coefficient was undefined. We used instead, where defined, the correlation coefficients between P6 and P7 of the resolved set for this part of the calculation.

## Results

We first discuss the resolution enhancement results of the methods individually using the diagnostic synthetic spectra, then consider their resolution enhancement performance on real glucose spectra, and finally we look at their use for 2D-COS and chemical contrast mapping of complex mammalian cell spectra.

### Synthetic Spectra

**Figure 2.**
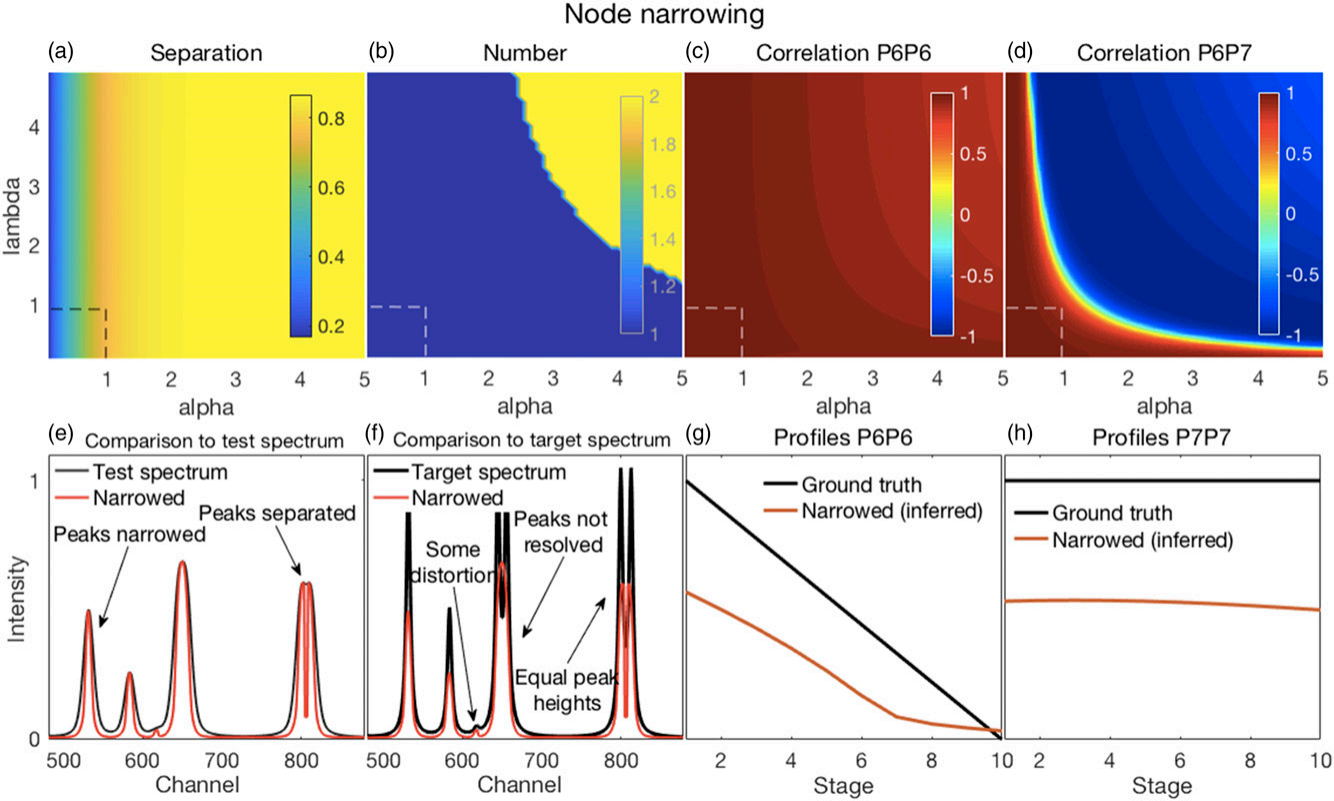
(a–d) Error surfaces obtained by varying the parameters selected for investigation. (a) Increases in alpha had a predominant effect on the separation between peaks in the filtered spectra with larger numbers showing more separation. (b) The number of peaks in the filtered spectra at variance with those present in the corresponding target spectra increased with increasing values of both alpha and lambda. (c) The overlapping P6–P7 peaks in the filtered spectra were not separated, thus the correlation coefficients shown are those between the P6 inferred in the filtered spectra and the P6 present in the target spectra. These showed a slight decorrelation with increasing values of alpha and lambda. (d) The correlation coefficients shown are those between P6 and P7, both inferred in the filtered spectra. A rapid loss of correlation and onset of an anti-correlation is observed with increasing values of alpha and lambda. (e–h) Plots show the most challenging spectrum subregion that best details the filtering performance for alpha and lambda both set to 1.0 (shown by the dashed lines in panels in a–d). (e) Filtering produced desirable peak narrowing and separation effects that are shown (red trace) relative to the starting spectrum (black trace). (f) Filtering also produced some ineffective peak separation and undesirable peak distortion results as shown (red trace) relative to the target spectrum (black trace). (g) The inferred P6 profile (because the peak was not resolved) in the filtered spectra was similar to its ground truth profile as (h) was the case for P7.

**Figure 3.**
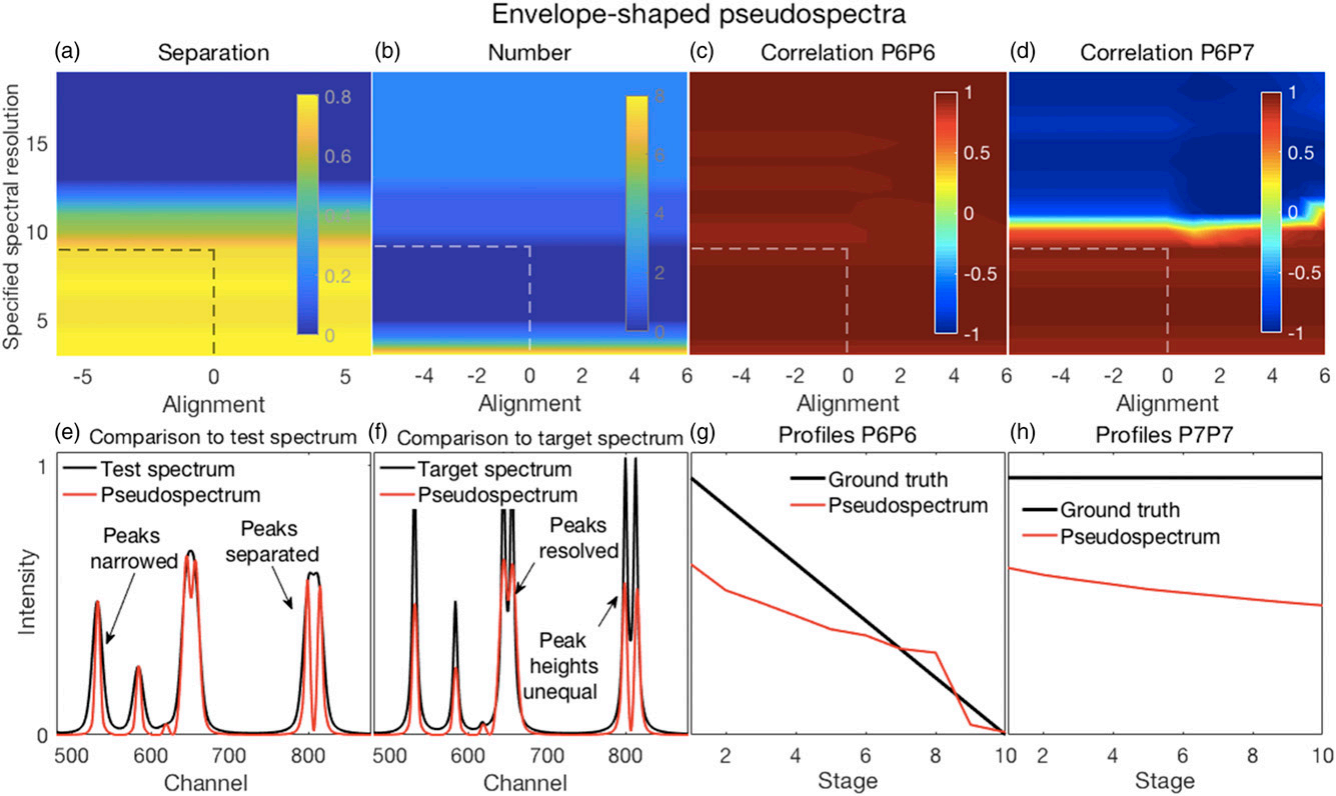
(a–d) Error surfaces obtained by varying the parameters selected for investigation. (a) Separation between peaks with larger numbers showing more separation. Smaller values of the specified spectral resolution produced greater separation between peaks. Alignments deviating from the zero position had little effect. (b) Smaller, but not minimal, values of the specified spectral resolution also produced an accurate number of peaks. (c) All correlation coefficients between P6 for both the target and pseudospectra were generally high. (d) Correlation coefficients between P6 and P7 of the pseudospectra changed rapidly with the specified spectral resolution, but from positive to negative and near the value corresponding to the truth set P1 (∼12 channels). Alignment had minor effects. (e–h) Plots show the most challenging spectrum subregion that best details the pseudospectra performance for a spectral resolution specified as nine and a shifted alignment with respect to the test spectra of zero as shown by the dashed lines in panels (a–d). (e) Pseudospectra had desirable peak narrowing and separation effects that are shown (red trace) relative to the starting spectrum (black trace). (f) Pseudospectra could also resolve the highly overlapping P6 and P7 peaks for smaller values of the specified spectral resolution but did not maintain equal heights for the P8 and P9 peaks. (g and h) Qualitatively similar relationships were observed for P6 and P7 profiles between their ground truths and the corresponding resolved peak profiles in the pseudospectra.

**Figure 4.**
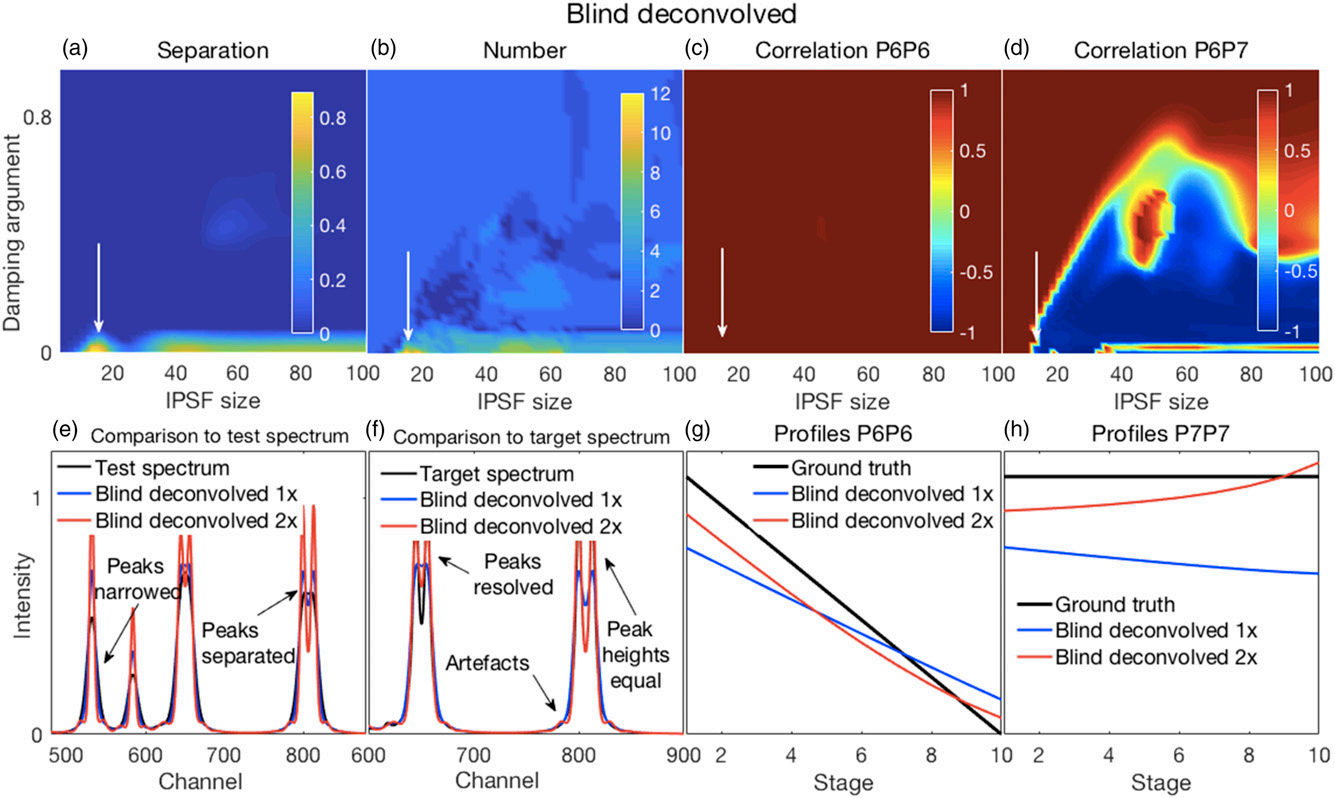
(a–d) Error surfaces obtained by varying the blind deconvolved 2× parameters selected for investigation. (a) IPSF sizes had little effect. Very small values of the damping argument produced greater separation between peaks. However, (b) they also produced an inaccurate number of peaks. (c) Correlation coefficients between P6 of the target and P6 of the deconvolved spectra were high for all parameter values. (d) Correlation coefficients between P6 and P7 of the deconvolved 2× spectra changed with adjustments in both parameters. (e–h) Plots show the most challenging spectrum subregion that best details the performance of blind deconvolution for an IPSF of 15 channels and a damping argument of zero as shown by the arrows in panels (a–d). (e) Deconvolved 1× spectra had modestly narrowed peaks (blue traces) with modest peak separation and both were intensified when deconvolved 2× (red traces). (f) Deconvolved spectra could not resolve the highly overlapping P6 and P7 peaks except when deconvolved 2×. However, this also produced artefacts. (g and h) Blind deconvolution produced very consistent results, thus very similar relationships were observed for P6 and P7 profiles between their ground truths and the corresponding resolved peak profiles in the blind deconvolved spectra. IPSF: instrument point spread function.

**Figure 5.**
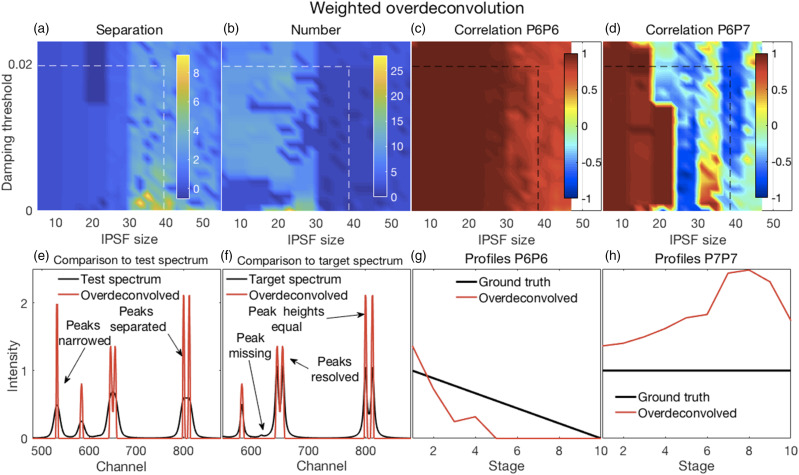
(a–d) Error surfaces obtained by varying the parameters selected for investigation. (a) Increased IPSF sizes produced better separation between peaks (larger numbers) and were (b) more likely to produce the correct number of peaks. The damping threshold had less of an effect. (c) Correlation coefficients between P6 of the target and P6 of the deconvolved spectra were high for all smaller IPSF sizes, but correlations tended to weaken with larger IPSF sizes. The damping threshold had less of an effect. (d) Correlation coefficients between P6 and P7 of the deconvolved spectra decorrelated sharply with IPSF sizes increasing above ∼20 channels with some noticeable effect of the damping threshold. (e–h) Plots show the most challenging spectrum subregion that best details the performance of over-deconvolution using an IPSF of 39 channels and a damping threshold of 0.02 as indicated by the dashed lines in panels (a–d). (e) Deconvolved spectra had very narrow peaks with clear peak separation. (f) The highly overlapping P6 and P7 peaks were clearly resolved with equal peak heights. However, the small peak at channel 618 was suppressed. (g and h) Over-deconvolution produced very inconsistent results on peak profiles, thus very dissimilar relationships were observed for P6 and P7 profiles between their ground truths and the corresponding resolved peak profiles in the over-deconvolved spectra. IPSF: instrument point spread function.

**Figure 6.**
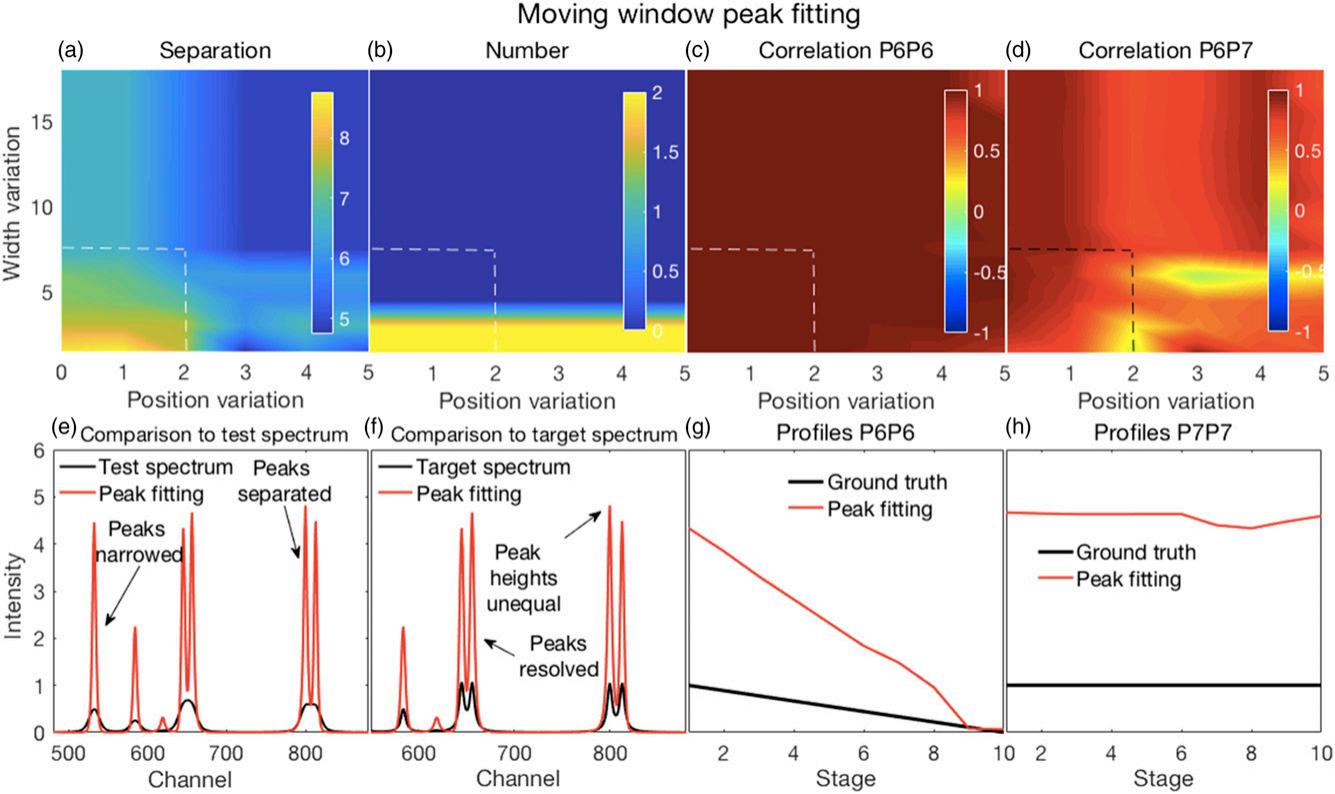
(a–d) Error surfaces obtained by varying the parameters selected for investigation and (e–h) plots showing the most challenging spectrum subregion that best details the moving window peak fitting performance. (a) Increases in permissible variations for both peak width and position, with larger numbers showing more separation, tended to reduce peak separation. (b) A correct number of peaks were counted in the fitted spectra (that is, the same as the number present in the target spectra) for allowable width variations exceeding the HWHM of P1. Position variations had no effect. (c) Correlation coefficients between P6 for both the target and deconvolved spectra were high everywhere; see also (g). (d) Though generally high, correlation coefficients between P6 and P7 of the deconvolved spectra showed more complex behavior. This suggested that peak fitting decorrelated these peaks for only a few values of the parameters. Fitted spectra, obtained with allowable peak and width variations up to 2 and up to 7.5, respectively (dashed lines in top panels), with peak widths reduced to 33% of those obtained in the fitting procedure (e) had high intensities and narrowed peaks with good peak separation; (f) could resolve the highly overlapping P6 and P7 peaks but with unequal heights, also true for the overlapping P8 and P9, compared to the target spectra. (g and h) Similar relationships were observed for P6 and P7 profiles between their ground truths and the corresponding resolved peak profiles in the fitted spectra.

**Figure 7.**
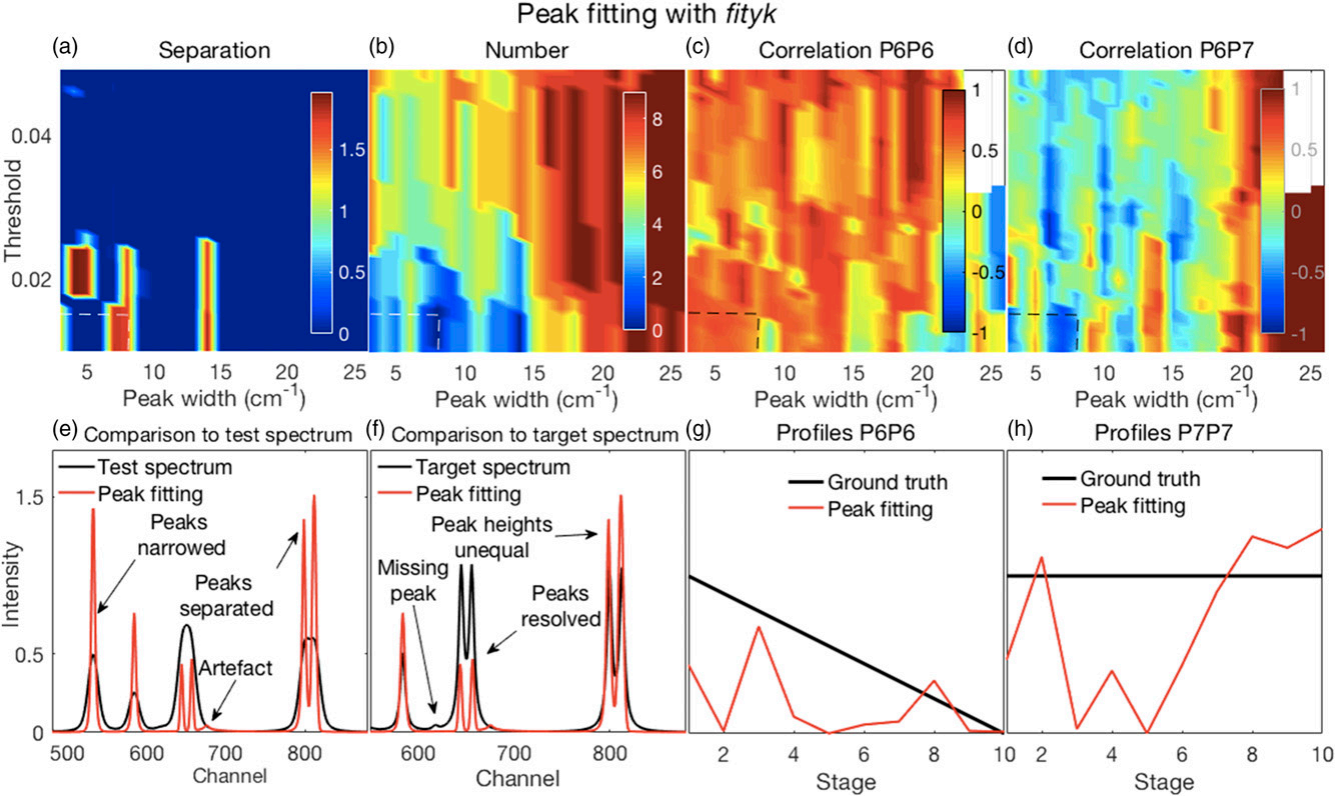
(a–d) Error surfaces obtained by varying the parameters selected for investigation and (e–h) plots showing the most challenging spectrum subregion that best details the Fityk performance. (a) Separation between peaks, with larger numbers showing more separation occurred in a few isolated regions where smaller amplitude thresholds and peak widths were specified. (b) A correct number of peaks were counted in the fitted spectra (that is, the same as the number present in the target spectra) in roughly the same regions where good peak separation occurred. (c) Correlation coefficients between P6 for both the target and deconvolved spectra were generally modestly high, though with uneven variations. (d) Correlation coefficients between P6 and P7 of the deconvolved spectra were generally weak or somewhat negative, except for large peak widths. Fitted spectra, obtained with thresholds and peak widths of 0.015 and 8, respectively (dashed lines in top panels), thereafter with peak widths reduced to 33% of those obtained in the fitting procedure (e) had high intensities and narrowed peaks with good peak separation but with unequal heights; (f) could resolve the highly overlapping P6 and P7 peaks but with reduced heights compared to the target spectra. (e and f) Artefactual peaks and peaks missing were noticed. (g and h) P6 and P7 profiles were very uneven.

In all of the figures shown in this section (Figs. 2–7), the *y*-axis labels for all the panels in the same row are shown in the left-most panel. Where used, the *x*-axis label “Stage” refers to the ten stages of change, for each of which a spectrum was created, as explained in the Methods section under Synthetic Spectra.

#### Node Narrowing

Illustrative results from applying the NN filter to the synthetic spectra are shown in Fig. 2 and a complete set of evaluation metric results are shown in Fig. S1 (Supplemental Material).

In general, filtering with NN produced the intended reduced peak widths and improved peak separation seen in Figs. 2a and e but failed to resolve the overlapping P6–P7 peak (Figs. 2b and f). However, the separated P8 and P9 peaks had equal heights, consistent with the target spectra (Fig. 2f). Filtering also caused some peak distortion (Fig. 2f). Another undesirable consequence was deviations of some filtered peak positions from their target spectrum positions (Figs. S1f and g).

Though unresolved, the filtered spectrum P6 and target spectrum P6 decorrelated slightly and somewhat commensurately with the amount of narrowing while the loss of correlation was much more rapid and turned negative for the caused filtered spectrum P6 and P7 (Figs. 2c and d). This was possibly caused when narrowing resulted in the inferred positions of P6 and P7 shifting further towards the respective tails of the narrowed, but unresolved, peak. P6 and P7 intensities at their inferred positions were thus less influenced by each other. Figures 2g–h show the profiles or trends at the inferred P6 and P7 positions of the filtered spectra, respectively, relative to the ground truth profiles of the target spectra. As is to be expected when peaks narrowed, gradual increases occurred in the root mean square error (RMSE) between filtered and corresponding target spectra (Fig. S1h). However, no rapid or abrupt changes were noted suggesting that no severe artifacts or distortions occurred.

Values of one have been recommended for both alpha and lambda^9^ and we concur that these (or slightly larger) values represent good choices for effective narrowing and separation while avoiding excessive distortions.

#### Envelope-Shaped Pseudospectra

Illustrative results of generating envelope-shaped pseudospectra are shown in Fig. 3 and a complete set of results are shown in Fig. S2. Overall, as shown in Figs. 3a–d and Fig. S2, varying the specified spectral resolution had more of an effect than changing the alignment, except when misalignment caused changes in peak positions affecting the number of peaks at incorrect positions and the sizes of the position errors (cf. Figs. S2f–h).

The EP method produced effective narrowing and peak separation, especially when specifying spectral resolutions similar to or less than the 12 channels of the truth set P1 (Fig. 3a). As all other peaks had widths less than that of P1, the smaller spectral resolutions were more consistent with the truth set features than larger spectral resolutions. Though these smaller spectral resolutions produced better results, excessively small resolutions (four channels or less) produced artefactual peaks (Fig. 3b). Hence, we chose a resolution of nine and an alignment of zero, the dashed lines in Figs. 3a–d for further analysis with related details in Figs. 3e–h. Figure 3e shows the narrowing effected on P4 and the separation of the overlapping P8 and P9 of the test set. Also shown in Fig. 3e is the resolution of the severely overlapped test sets P6 and P7, and in Fig. 3f, the relationship of the resolved peaks to the corresponding peaks in the target set. Resolving the fused P6–P7 peak also produced the correct number of peaks in the pseudospectra (Fig. 3b). However, Fig. 3f also shows that P8 and P9 were separated, but with unequal heights, thus producing an undesirable effect. Though P6 and P7 were resolved, and their profiles resembled those of the corresponding truth set profiles (Figs. 3g and h), they were both downward sloping. Thus, the pseudospectrum P6 was correlated with the truth set P6 (Fig. 3c) and the pseudospectrum P6 and P7 remained correlated (Fig. 3d).

Despite the choice of parameters made above, the optimal choice of parameters might be dependent on the application because, for the chosen parameters, many peaks had incorrect positions (Fig. S2f), albeit with small errors (Fig. S2g).

#### Blind Deconvolution

Blind deconvolution produced very modest effects on the test spectra (see Fig. S3A) and subsequently we investigated a repeated deconvolution where a second deconvolution was applied to the results of the initial deconvolution (see Fig. S3B). The broad IPSF estimates obtained from a single blind deconvolution (BD 1×) became narrower after a repeated blind deconvolution (BD 2×), consistent with a BD applied to better resolved spectra (Fig. S4). The RMSE for the BD 2× (Fig. S3Bh) showed a global minimum near damping argument values of zero and IPSF sizes around 15 channels. We chose this region for detailed examination. Illustrative results for blind deconvolved 2× are shown in Figs. 4a–d and related details in Figs. 4e–h.

The damping argument in the BD algorithm is intended to suppress ringing or satellite peaks that can emerge during deconvolution. Thus, higher values of the damping argument appeared to suppress peak separation (Fig. 4a) while also suppressing the emergence of artefactual peaks (Fig. 4b). The correlations between P6 of the deconvolved spectra and that of the target spectra were mostly uniform, except for a narrow region of very small damping arguments where complex changes for IPSF sizes were evident. A more complex partition of the correlation space was observed for the correlations between P6 and P7 of the deconvolved spectra indicating that deconvolution also produced decorrelations and inverted correlations for these peaks.

Figures 4e and f show the increased peak narrowing obtained with the second BD (red trace) of the already deconvolved spectrum (blue trace) that also resulted in good separation with equal heights of P8 and P9 and a resolution of the fused P6 and P7. However, it also resulted in artefactual or satellite peaks, thus increasing the number of “surplus” peaks in these spectra for very small damping argument values (Fig. 4b). The peak profiles of the resolved P6 and P7 in the deconvolved spectra were smooth and monotonic (Figs. 4g and h). However, repeated deconvolution produced an accentuation of the slight curvatures present in the profiles of a single deconvolution, thus causing greater deviations from the corresponding profiles of the truth set.

Thus, trade-offs exist between the various performance measures and the selection of a single or repeated deconvolution will depend on the application. For example, if good correlations need to be estimated between measured spectra and the corresponding sample ground truths, a single blind deconvolution might be appropriate, though this will come at the expense of a reduced, or possibly inadequate, resolution in the deconvolved spectra.

#### Weighted Over-Deconvolution

Illustrative results obtained with WO are shown in Figs. 5a–d and detailed analyses in Figures 5e–h. A complete set of results are shown in Fig. S5.

Over-deconvolution could produce very good separation between peaks (Fig. 5a) consistent with very narrow resolved peaks (Fig. 5e). Over-deconvolution could also produce the correct number of peaks was (Fig. 5b). Particularly important in these investigations were an assessment of the resolution of fused peaks. The fused P6 and P7 peak was effectively resolved with over-deconvolution (Figs. 5e and f). Profiles were somewhat decorrelated for the resolved P6 and truth set P6 and sharply decorrelated for the resolved P6 and P7 for some IPSF sizes. This was due to rather erratic peak heights between stages that were obtained with over-deconvolution as can be seen in Figs. 5g and h. These effects were mostly due to increases in the IPSF size where relatively sharp transitions occurred around 30 channels. The damping threshold had little effect, suggesting that the major damping of satellite peaks occurred due to the weighting scheme. However, suppression of artefactual peaks could also cause the elimination of small true peaks (Fig. 5g).

#### Moving Window Peak Fitting

Illustrative figures of merit and details for fitted spectra, with their peak widths further reduced to 33% of the fitted widths with commensurate increases in peak heights, are shown in Fig. 6.

The results shown in Figs. 6a–d, and also in Fig. S6, are dependent on the percentage reduction in peak width that could be specified when implementing the algorithm. Thus, these results could change due to such specifications. Nevertheless, they show the general trends that could be expected. Given a specified reduction of peak width to 33% of the fitted widths, a minimum was observed in the number of peaks with inaccurate positions for allowed width variations of 1.25 times the P1 FWHM of six channels, thus 7.5 channels, and peak positions that could vary up to two channels to either side of the starting positions (see Fig. S6). Spectra obtained for these conditions, shown by the dashed lines in Figs. 6a–d, were further analyzed as shown in Figs. 6e–h.

In contrast to peak separation shown in Fig. 6a, the number of correct peaks shown in Fig. 6b was not dependent on both parameters, but only on the peak width variation. The correlation coefficients between the resolved P6 and the target P6 (Fig. 6c) remained high for all values of the parameters. Similar trends can be seen in Fig. 6g. A relatively high correlation coefficient was observed between the resolved P6 profile and the resolved P7 profile (Fig. 6d) though the resolved P7 profile resembled much more the truth set P7 profile (Fig. 6h). This was caused by a slight decline in the resolved P7 trend and scaling by the standard deviation. Figures 6e and f show details of the resolved spectra. As mentioned, the fitted peaks could be narrowed as desired, up to a width of one channel. As a result, peak separation can be improved, and peak heights increased. Peak fitting also resolved the overlapping P6 and P7. However, the resolved peaks did not have accurate relative peak heights or smooth profiles (Figs. 6g and h).

#### Peak Fitting with Fityk

Illustrative figures of merit and details for fitted spectra, with their peak widths further reduced to 33% of the fitted widths with commensurate increases in peak heights, are shown in Figure 7. Further details are shown in Fig. S7.

As for the MF method, the results shown in Figs. 7a–d, and also in Fig. S7, are dependent on the percentage reduction in peak width that was specified as 33% of the fitted widths. Smaller specified initial peak widths yielded reduced final peak widths. With larger specified initial widths, overlapping peaks were not separated and peaks were missed. This affected the separation between peaks with more separation occurring in a few isolated regions where smaller peak widths (and amplitude thresholds) were specified (Fig. 7a). This also affected the number of correct peaks (Fig. 7b). Correlation coefficients between P6 for both the target and deconvolved spectra shown in Fig. 7c were generally modestly high, though with uneven variations consistent with their uneven profiles as evident in Fig. 7g. Correlation coefficients between P6 and P7 of the deconvolved spectra were generally weak or somewhat negative, except being strongly positive for large peak widths consistent with not being separated. The details in Figs. 7e and f of narrowed fits obtained with a threshold of 0.015 and an initially specified peak width of 8 channels showed effective narrowing and peak separation, but also that some peaks were missed, the presence of artefacts and uneven heights leading to uneven peak profiles as evident in Figs. 7g and h.

### Glucose Spectra

**Figure 8.**
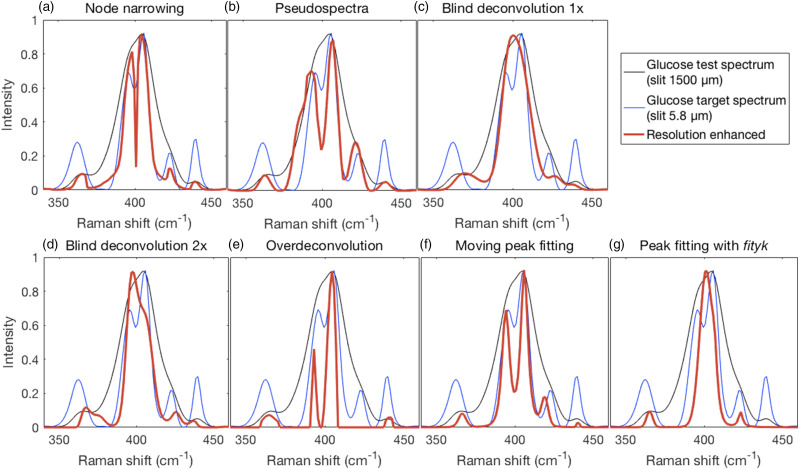
Results of the resolution enhancement methods (red traces) on the wide-slit glucose test spectrum (black traces) compared to the narrow-slit target spectrum (blue traces). Test, target, and resolution enhanced traces in the shown region were scaled to the same maximum intensity except in the first two panels where the resolution enhanced traces were constrained by the applied methods. (a) Node narrowing produced the correct number of peaks at good peak positions, but with some distortion of peak shape. (b) Likewise, for pseudospectra. (c) Neither blind deconvolution nor (d) repeated blind deconvolution resulted in a resolution of the fused peaks though the latter resulted in the emergence of a clear shoulder. (e) Over-deconvolution resolved the fused peaks with reasonably modest relative intensities but missed one peak. (f). The narrowed moving window fitted spectrum showed a resolution of the fused peaks with good relative intensities. (g) The narrowed spectrum generated from peak fitting with Fityk missed one peak and did not resolve the fused peaks.

All of the resolution enhancement methods were applied to the glucose test spectrum (slit at 1500 μm) and compared to the glucose target spectrum (slit at 5.8 μm). Shown in Fig. 8 are details of unassigned skeletal and torsional modes^60^ where two adjacent peaks near 400 cm^−1^ are fused in the test spectrum but resolved into two peaks of differing heights in the target spectrum. Table I shows semi-quantitative results for the peak positions of resolution enhanced spectra that were resolved in the 5.8 μm high resolution spectrum.

**Table I.** Peak positions for resolution enhanced spectra and their absolute deviations relative to the resolved high-resolution peaks shown in Fig. 8.

Method	cm^−1^	cm^−1^	cm^−1^	Mean	Standard deviation
**Narrow slit**	**362.6**	**422.3**	**439.9**	—	—
NN	365.2	422.3	438.7	1.2667	1.3013
EP	363.9	421.0	439.9	0.8667	0.7506
BD 1×	370.3	426.1	437.4	4.6667	2.7062
BD 2×	367.7	426.1	437.4	3.8000	1.3000
OD	365.2	—	441.2	—	—
MF	366.4	418.5	439.9	2.5333	2.1939
FF	365.5	423.5	—	—	—

Spectra obtained with NN and envelope-shaped pseudospectra are constrained by the intensities of the test spectra as shown in Figs. 8a and b because the spectral intensities of the processed spectra (red traces) peak maxima fall on or within the intensity envelope of the test spectrum (black traces). Thus, they cannot be expected to conform to the relative peak heights of the target spectrum but can be assessed based on their ability to resolve the fused peaks and maintain good positions for narrowed peaks. In contrast to the synthetic test spectra (Figs. 3f and 4f), NN resolved the fused peaks near 400 cm^−1^. The pseudospectra also resolved these peaks. Both also produced reasonable peak positions. Blind deconvolution, either 1× (Fig. 8c) or 2× (Fig. 8d), was not able to resolve the fused peaks though the latter resulted in the emergence of a clear shoulder. Both produced inaccurate peak positions and a false peak (∼375 cm−^1^) occurred in the 2× case. Note also here an inconsistent performance relative to the synthetic test spectra (Fig. 4f). Over-deconvolution (Fig. 8e) resolved the fused peaks with good relative intensities, but not others. It also did not detect one peak. The most successful performance on these data was the moving window fitting procedure where the fused peaks were resolved, all peaks were placed well, but all the relative peaks’ heights? did not match well those of the target spectrum. Both over-deconvolution and fitting produced results more consistent with those obtained on the synthetic test spectra (Figs. 5f versus 8e and 6f versus 8f, respectively). Fityk fitting (Fig. 8g), with fitted peaks subsequently narrowed, did not resolve the fused peaks near 400 cm^−1^ and one peak was missed, but the positions of the remaining smaller peaks were good.

### CHO Cell Spectra

**Figure 9.**
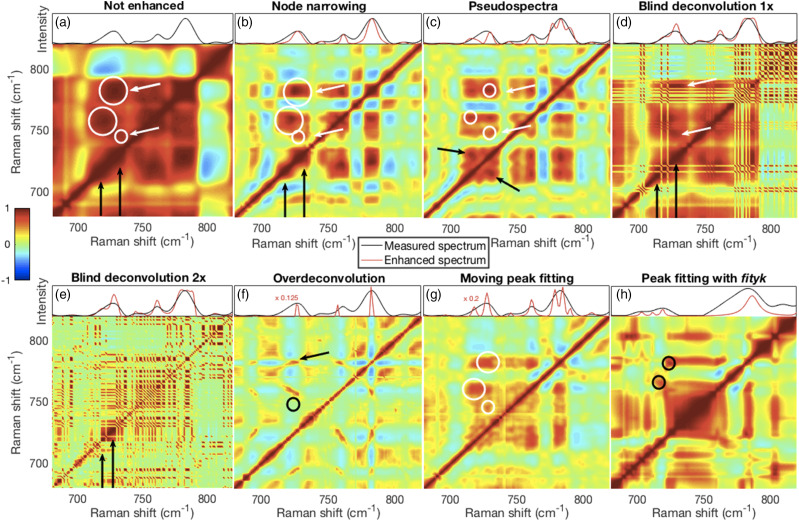
Details of 2D-COS correlation coefficient maps are shown in the lower portions of panel assemblies (a)–(h). The top portion of each panel shows a corresponding section of the first spectrum in the set (not enhanced, black trace) and its resolution enhanced spectrum (red trace) for the respective methods. Additional details are described in the main text. (a) Unenhanced spectra show broad correlation features. (b) NN did not resolve the fused phosphatidylcholine (∼717 cm^−1^) and adenine (∼725 cm^−1^) peaks (top portion) but did lead to clear separation of the correlation features. (c) The resolved phosphatidylcholine and adenine peaks in the pseudospectrum are visible in the top portion of the panel and a clear separation of the correlation features in the bottom portion. (d and e) Both blind deconvolution approaches produced narrowed and also small artefactual peaks, the second pass method (e) more so, and these are visible in the top portions of panels (d and e) causing the fragmented correlation features in the bottom portions. (f) The very narrow over-deconvolved peaks produced sharp correlation features, but also missed some peaks while (g) narrowed moving fit peaks were well resolved but with inconsistent correlation features. (h) Narrowed peaks fitted with Fityk, for the limited spectra analyzed, showed resolution of the phosphatidylcholine and adenine peaks (left and right black circles, respectively), but also a broad central feature that appeared related to the extensive tails of fitted spectra. 2D-COS: two-dimensional correlation spectroscopy; NN: node narrowing.

The performance of the various methods was examined on 2D-COS using the 462 spectra obtained from a dry-fixed CHO cell. Details of the correlation coefficient maps from 680 to 820 cm^−1^ are shown in the lower portions of panels (a) to (g) in Fig. 9. The top portion of each panel shows the intensities of the corresponding representative spectrum (not enhanced, black trace) and their resolution enhanced spectrum (red trace). These spectra were normalized to the intensities of their tallest peaks. Although asynchronous spectra might provide more of a resolution enhancement effect,^8^ synchronous spectra are arguably more in need of better resolution. They are also more readily interpreted and are more often used. Therefore, we have used synchronous spectra for evaluating the resolution enhancement effects of the different methods.

Despite producing broad correlation features (Fig. 9a), unenhanced spectra nevertheless hinted at the existence of overlapping phosphatidylcholine and adenine peaks shown by the “bulges” on the diagonal near 717 cm^−1^ and 725 cm^−1^ (black arrows) and the slightly offset correlation intensity maxima (white circles). The laterally displaced positions of the large circles and the lack of cross diagonal peaks near the black arrows suggested that the phosphatidylcholine and adenine peaks were not highly correlated with each other. However, one could infer from the color (correlation strength) that the thymine peak near 746 cm^−1^ (small circle and lower white arrow) was related to the composite nucleic acid peak near 783 cm^−1^ (upper white arrow). Thus, these results demonstrate the capability of 2D-COS to enhance the spectral resolution of peaks even when they are not resolved in the individual spectra.^8,61,62^

Node narrowing (Fig. 9b) produced similar results to the unenhanced spectra even though the fused phosphatidylcholine and adenine peaks were not resolved in the enhanced spectrum (red trace, top portion of panel). However, narrowing caused more discrete and interpretable correlation features than the unenhanced spectra. Pseudospectra evinced a resolution of the fused phosphatidylcholine and adenine peaks (Fig. 9c, top portion) and greater separation (Fig. 9c, bottom portion) than NN between the correlation features (top two white circles). However, in contrast to the unenhanced and NN cases, the cross-diagonal peaks indicated by the black arrows suggested that the phosphatidylcholine and adenine peaks were incorrectly highly correlated. Also, some correlation between thymine (bottom circle and white arrow) and other nucleic acids (top circle and white arrow) was observed.

Though not clearly resolved, the BD red traces did produce evidence of separate phosphatidylcholine and adenine peaks (Figs. 9d and e). A relationship was also visible in the single pass blind deconvolved spectrum between thymine and other nucleic acids as indicated by the white arrows (Fig. 9d). However, the fragmentation caused by artefactual peaks made the correlation coefficient maps for both approaches difficult to interpret. Over-deconvolution yielded very sharp peaks that produced clearly defined correlation features (Fig. 9f). Some of these were also slanted suggesting that their positions shifted. The phosphatidylcholine peak near 717 cm^−1^ and thymine peak near 746 cm^−1^ were missing while broad features around 700 cm^−1^ were observed. Thus, no correlation between thymine (black circle) and other nucleic acids (black arrow) was indicated.

Peaks observed with the moving window peak fitting procedure could be arbitrarily reduced (here to 33% of fitted peak widths) and seemed to generate well-resolved spectra (Fig. 9g, top). However, the correlation maps (Fig. 9g, bottom) were poorly resolved and inconsistent with well-resolved spectra. Though Fityk is relatively easy to use in an interactive mode, we had difficulty processing all 462 spectra in a semi-automated manner. Peaks fitted with Fityk on a subset of the spectra (196 to 218) and subsequently narrowed to 33% of their fitted widths did show a separation of the phosphatidylcholine and the adenine peaks (black circles).

Details of the profiles obtained by the different methods for the phosphatidylcholine peak near 717 cm^−1^, of spectra 196 to 218 inclusive, are shown in Fig. S8. The correlation coefficients between these segments of the measured and resolved spectra are also shown for each method. If the phosphatidylcholine peak was resolved in the mean spectrum of the entire set of 462 spectra, the position of the maximum or center of the peak was used, otherwise the maximum or channel with shift closest to 717 cm^−1^ was used. High correlations are not necessarily desirable because the extent of resolution of overlapping, but unrelated, peaks should commensurately decorrelate them.

Segments of the 695 to 810 cm^−1^ mean measured and mean resolved spectra are shown in Fig. S9. Also shown are the RMSE values between the complete mean measured and mean resolved spectra. Increased resolution, thus narrower peaks, will inevitably increase the RMSE values. Higher RMSEs will also occur with the deconvolution and fitting methods. RMSE values will also increase with aberrant results such as the large artefacts visible in the BD 2× spectral segment (Fig. S9d).

**Figure 10.**
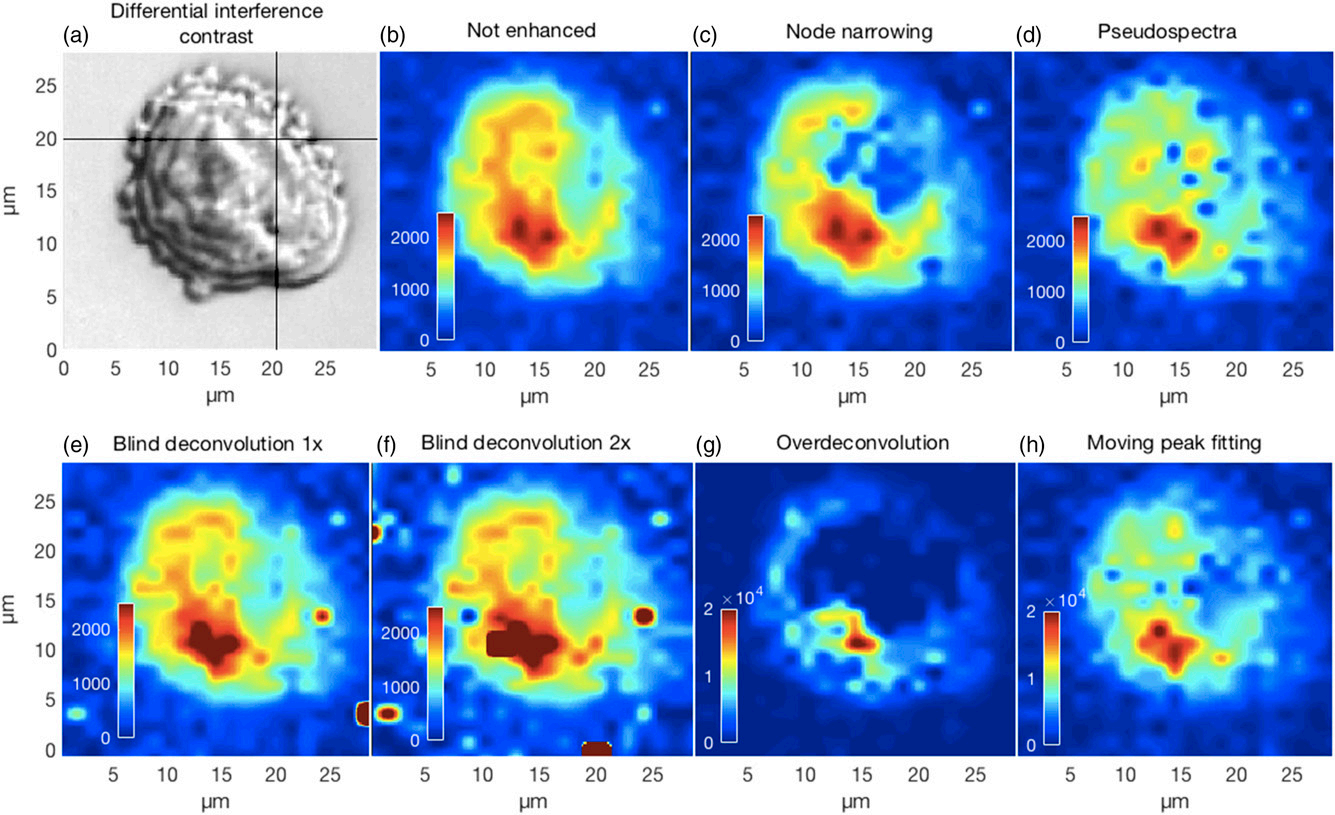
(a) Differential interference contrast image of a CHO cell fixed in 0.09% (10% physiological concentration) saline and air dried. Panels (b–h) show the differences in the chemical contrast images for the lipid phosphatidylcholine peak near 717 cm^−1^ obtained for the various resolution enhancement methods where the full set of spectra could be processed.

Effective resolution enhancement and the associated decorrelation should produce peaks that are not affected by the intensities of unrelated neighbors. This would improve the contrast of chemical images by showing more accurate distributions and intensities of specific chemical moieties. In Fig. 10 we show the results of mapping the 717 cm^−1^ phosphatidylcholine lipid peak for the CHO cell in panel Fig. 10a. Though all methods show maximum intensities near the lower, slightly left, part of the image, the particulars differ. For example, the over-deconvolved lipid peak is more distributed around the periphery of the cell compared to the other methods. This example suggests that decorrelating overlapping or fused peaks can lead to an apparent improvement of the spatial resolution in chemical contrast images. Though outside the scope of the current work, these results also suggest that an assessment of the resolution enhancement accuracy could be obtained by Raman spectroscopy followed by various fluorescent stains.

### Discussion

We have evaluated six resolution enhancement methods along various dimensions and discuss the major findings, the limitations of our study, and the need for ongoing specific research below. We have refrained from giving strong guidance on selecting any of the methods for automated or semi-automated use because of the findings, the difficulties of implementation and the limitations of the study that we also discuss below. Nevertheless, the presented results, as well as the Table II summary, might guide readers towards using one or another method. If so, we suggest that readers apply adequate caution when proceeding.

**Table II.** Qualitative overall summary of the results obtained from the methods investigated.

Name (type)	Parameters	Resolution enhanced?	Peaks separated?	Peak artefacts	Correlation recovery^a^
Node narrowing (derivative, constrained)	2	Yes	Some	False, missing, distorted	Not applicable^b^
Envelope-shaped pseudospectra (derivative, constrained)	4	Yes	Some	False, missing, distorted	Not applicable^b^
Blind deconvolution 2x (deconvolution)	3	Yes	Some	False, missing, distorted	Good
Weighted over-deconvolution (deconvolution)	2	Yes	Yes	False, missing, distorted	Poor
Moving window peak fitting (simultaneous peak fitting)	7	Yes	Yes	Distorted	Good
Fityk (fit-and-subtract peak fitting)	4	Yes	Some	False, missing, distorted	Poor

^a^On synthetic spectra.

^b^Constrained to starting spectrum and cannot differ except due to artifacts.

#### Our Major Findings

First, all the methods succeeded in effecting some degree of peak narrowing with concomitant separation between peaks. Second, they were all capable of resolving some highly overlapped peaks. Third, the peak heights of resolved spectra differed fundamentally for these methods because the intensities obtained by NN and pseudospectrum generation are constrained to those of the starting spectra. Fourth (data not shown), deconvolution methods occasionally produced very bad and unexpected results. Fifth, the NN, pseudospectrum generation, and BD methods were relatively fast while the over-deconvolution and peak fitting methods were much slower. Sixth, the method performances were variable depending on their parameter settings and the spectra processed. Finally, even with fixed and arguably good parameter settings, there was considerable variability within the methods. In general, they seemed to be sensitive to slight variations in the spectra that might have been due to residual measurement variations after preprocessing. Table II summarizes the qualitative interpretation of the results.

#### Limitations of This Study

The variable performances made an assessment of the methods quite difficult, and we tried to address this by surveys of some parameter setting-induced error surfaces. Nonetheless, it was clear that good parameter settings for some spectra did not transfer well to other spectra—irrespective of moving between synthetic and real spectra or within these categories. As it was not feasible to examine the effects of all parameters and thresholds, a further difficulty arose in determining such settings so as to be reasonably consistent across all methods. Such a problem occurred, for example, with setting thresholds to suppress artefactual peaks.

Some decisions also had to be made in calculating the various performance measures. Thus, would a fused peak be counted as a single peak or two peaks that overlap? If counted as one, as we did, the other one is “missing”, and the determination of a mean peak shift is dependent on the contribution of the distance between the position of the fused peak and that of the nearest peak of the resolved pair in the truth set. Associated with this was the fact that some measures, normally used to estimate the “goodness” of a result, would give a worse reading with a better result. For example, though the RMSE might increase due to missing, shifted, or artefactual peaks, it also increases with resolutions better than that of the target data.

Our analyses also had their shortcomings due to reasonable length limitations. They illustrated how making an assessment of the methods must address many challenges, in particular when making judicious choices to reduce the complexity of the challenges to manageable levels. For example, only one resolved spectrum of a set, consistent for all methods, was used for detailed examination while others might have yielded different results. Perhaps most helpful was our use of synthetic spectra with known ground truths. Due to all the above limitations, therefore, it is important to see our analyses not in terms of a competition; we expect that routine practitioners of the existing methods will be able to improve on these results.

#### Looking Ahead

The variable performances of the alternative resolution enhancement methods investigated highlighted the importance of this type of study. Our analyses of both synthetic and real spectra should most of all provide useful insights into the characteristic aspects of the different approaches, as well as be most relevant to progress towards automated spectral analysis. Indeed, further research is crucial to improve peak resolution methods to achieve the full promise of Raman spectroscopy analysis of mammalian cells as can be seen in Figs. 9 and 10.

Given the variable performances of these methods, what might be their most problematic shortcomings and how could these be remedied? We address four issues of concern, starting with the issue of missing or artefactual peaks and thereby the difficultly of establishing the correct number of peaks in a real spectrum. Methods not using deconvolution usually depend on derivative approaches to find the peaks and their positions in a spectrum. In practice, some fused peaks cannot adequately be resolved by derivatives and going beyond second derivatives might generate artefactual peaks due to residual low frequency noise in smoothed spectra. Fitting-and-subtracting methods^49,63^ might also be of little use because two or more fused peaks can often be fitted quite well with a single peak. With deconvolution methods, artefactual peaks tend to be the main problem. Currently, thresholds and weighting schemes are used to combat them. However, these are prone to yield unsatisfactory results and perhaps radically different approaches should be developed.

Second, establishing correct peak positions is likewise essential but problematic. It is clear that this problem is somewhat entangled with the preceding one, especially where a method might rely on, or use, starting values obtained separately. One possibility involves exploiting latent information in the hyperspectra as part of an iterative resolution enhancement process. Thus, some information about peak positions, for example, could be extracted from the hyperspectra using second derivatives,^50^ and potentially PCA, MCR, or other methods. This might lead to hyperspectra with improved resolution from which more accurate peak positions can then be extracted. The procedure is iterated until a convergence criterion is reached. However, whether multivariate methods such as PCA can be used to effectively determine in complex spectra peak positions in an automated manner still needs to be investigated.

Third, the most critical issue for correlation-based methods is correct peak heights. Because node narrowed and pseudospectrum peak heights are constrained by the initial spectrum, peak height variations are, in principle, less of a problem. However, for precisely this reason, even for resolvable fused peaks, the resolved peaks might only be minimally decorrelated whereas effective correlation-based analyses will benefit from strong decorrelation such that the resolved peaks exhibit close to their true profiles. The other methods seem to produce peak heights that were inconsistent with those expected for either the synthetic or the real glucose spectra. It is not presently clear how to address these effects; some investigations into fundamental aspects of these methods might be required.

Finally, adapting methods for automated implementation is needed to enable more practical, high-throughput and reliable applications of Raman spectroscopy. Where large numbers of spectra are to be processed in a fully automated manner, a method cannot be chaperoned for optimal processing of individual spectra. How should parameters then be set or chosen in automated procedures as well as implemented automatically? This becomes a critical issue for inhomogeneous hyperspectral data, such as intracellular spectra or spectra from semiconductor devices where adjacent spectra could be substantially different. Though we have previously developed a fully automated peak fitting method based on a fit-and-subtract approach to find good starting peak parameters followed by a gradient descent method to optimize them, its current implementation is too slow for use with large numbers of spectra,^63^ especially if large numbers of cells are to be analyzed. Chen and Garland^50^ devised a method to fit Pearson VII distributions to large sets of infrared absorption spectra, and this is to the best of our knowledge the only other method potentially addressing automation for such applications. Their method used peak parameters that were obtained from a second derivative approach to determine peak positions, peak widths were estimated based on the system under study, shapes assessed to be near Lorentzian and amplitudes were algebraically solved given initial values for the other parameters. The method then optimized peak parameters with the simplex optimization method from these starting parameters. To obtain good starting parameters, a spectrum with sufficient information to provide adequate estimates needs to be chosen. Optimized parameters for this spectrum then serve as starting estimates for the adjacent spectrum, and this is repeated iteratively to fit the entire array of spectra. However, full automation in this case would require methods to select the best spectrum for determining the initial peak parameters, in a fully automated manner, and to accommodate heterogenous spectra. Perhaps artificial intelligence approaches could be deployed to address these tasks. Because this is a peak fitting method, band parameters can be artificially narrowed as done for the other peak fitting methods (with commensurate height adjustments) to effect resolution enhancement.

## Conclusion

All the methods succeeded in effecting some degree of peak narrowing with concomitant separation between peaks and in resolving some highly overlapped peaks. However, their performances were quite variable and dependent on their parameter settings and the spectra processed, thus making automated implementations yet infeasible.

Taken together, what are the most serious impediments observed for adequate fully automated or semi-automated performance on Raman spectra? For the narrowing methods (NN, envelope-shaped pseudospectra), the number of peaks and their positions are determined with derivatives; hence, this is an inherent limitation to better performance. For the deconvolution methods, the implementation of the IPSF is critical, either by defining its size (blind deconvolution) or more extensive aspects that include its lineshape (over-deconvolution). The generation of false peaks is also a problem. Peak fitting methods (moving window peak fitting, Fityk) are highly dependent on knowing the number of peaks present and their approximate positions. Addressing all these issues should facilitate the use of these methods for hyperspectral data.

Considering the use of the methods as they currently are, some suggestions follow. Where the need is limited to reducing peak widths to improve analysis or for presentation, NN or pseudospectra could be used. In fact, using them jointly might be useful as well as feasible since they are both quite fast and seem to have some complementary performance. For both improved resolution and correlation analysis, deconvolution methods could be useful, but improvements in performance, especially consistency, are needed. Overall, the peak fitting approaches seem to offer the most promise though they will need to have improvements in consistency and peak height estimates and to be adapted in various ways, for example, improved processing times, to make the analysis of large numbers of spectra feasible. Furthermore, having parameters for individual peaks in a set of spectra affords much flexibility in subsequent analyses, something not offered by other approaches.

Can we do better? We hope that these insights might spur further research by virtue of having highlighted the need for effective resolution enhancement approaches, and in particular those aspects that need improvement.

## Supplemental Material

sj-pdf-1-asp-10.1177_00037028211061174 – Supplemental Material for Critical Evaluation of Spectral Resolution Enhancement Methods for Raman Hyperspectra Supplemental Material, sj-pdf-1-asp-10.1177_00037028211061174 for Critical Evaluation of Spectral Resolution Enhancement Methods for Raman Hyperspectra by H. Georg Schulze, Shreyas Rangan, Martha Z. Vardaki, Michael W. Blades, Robin F. B. Turner and James M. Piret in Applied Spectroscopy
