# Supplementary material for: Critical Evaluation of Spectral Resolution Enhancement Methods for Raman Hyperspectra
Source: Appl Spectrosc. 2021 Dec 22;76(1):61–80. doi: 10.1177/00037028211061174 (PMC8750138; doi:10.1177/00037028211061174)
Supplement: sj-pdf-1-asp-10.1177_00037028211061174 – Supplemental Material for Critical Evaluation of Spectral Resolution Enhancement Methods for Raman Hyperspectra [file sj-pdf-1-asp-10.1177_00037028211061174.pdf]

Critical Evaluation of Spectral Resolution Enhancement Methods for Raman Hyperspectra

(Supplementary Material)

H. Georg Schulze,[1] Shreyas Rangan,[2,3] Martha Z. Vardaki,[4] Michael W. Blades[5],

Robin F. B. Turner,[2,5,6*] and James M. Piret[2,3,7*]

1. Monte do Tojal (Caixa Postal 128), Hortinhas, Terena (São Pedro), 7250-069, Portugal.
2. Michael Smith Laboratories, The University of British Columbia, 2185 East Mall, Vancouver, BC, Canada, V6T 1Z4.
3. School of Biomedical Engineering, University of British Columbia, 2222 Health Sciences Mall, Vancouver, BC, Canada, V6T 1Z3.
4. Department of Medical Physics, School of Health Sciences, University of Ioannina, 45110 Ioannina, Greece.
5. Department of Chemistry, The University of British Columbia, 2036 Main Mall, Vancouver, BC, Canada, V6T 1Z1.
6. Department of Electrical and Computer Engineering, The University of British Columbia, 2332 Main Mall, Vancouver, BC, Canada, V6T 1Z4.
7. Department of Chemical and Biological Engineering, The University of British Columbia, 2360 East Mall, Vancouver, BC, Canada, V6T 1Z3.

[*]Corresponding Authors (*turner@msl.ubc.ca* and *james.piret@ubc.ca*)

Here we show some explanatory lines of code for the weighted overdeconvolution method, as well as more detailed and complete results for the various resolution enhancement methods.

Weighted overdeconvolution. As explained in the main text, an algorithm using the MATLAB "fmincon" optimization routine was used to deconvolve spectra with progressively larger IPSFs to force narrower peaks. The spectrum to be processed was also used to provide starting values for the procedure. These were obtained by shaping the squared values of the input peaks to their corresponding input values, thus providing narrow starting values. A weighting scheme was used to suppress satellite bands. The weights consisted of the input spectrum normalized to its maximum value. The deconvolved spectrum was reconvolved with the IPSF and the 2-norm of the difference between input and reconvolution, element-wise multiplied with the weighting term, was used as the cost function to minimize. Explanatory lines of code follow.

```
indices = deconvolved_spectrum < damping_threshold;
deconvolved_spectrum (indices) = 0.0;
reconvolved_spectrum = conv(deconvolved_spectrum,IPSF,'same');
weights = (input_spectrum/max(input_spectrum));
     fval = norm((reconvolved_spectrum –
input_spectrum).*weights,2);
```

Node narrowing. Results for the synthetic test spectra filtered with the node narrowing filter are shown in Figure S1.

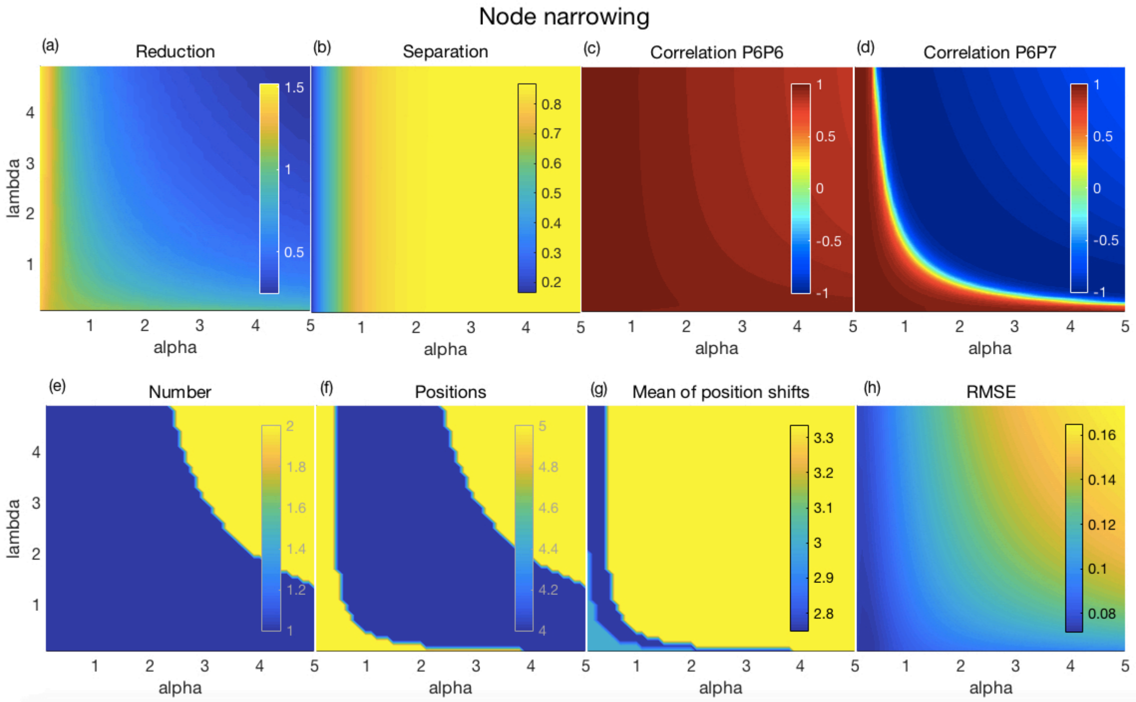

Figure S1. (a) Reduction in peak width with smaller numbers indicating larger reductions. Increases in both lambda and alpha produced reduced peak widths. (b) Separation between peaks with larger numbers showing more separation. Increases in alpha had a predominant effect, overriding the effects of reduced peak widths due to increases in lambda. (c) High correlations were observed between P6 for the target and filtered spectra with a tendency to diminish slightly with increasing values of alpha and lambda. (d) In contrast, correlations between P6 and P7 of the filtered spectra, initially high due to overlap, declined rapidly to anti-correlations with increasing values for alpha and lambda. (e) The difference between the number of peaks counted in the filtered spectra and those present in the target spectra increased with increasing values of alpha and lambda. (f) The number of peaks in the filtered spectra with positions at variance with their corresponding positions in the target spectra showed a minimum for intermediate values of alpha and lambda and (g) the extent of variation tended to increase with increasing values of alpha and lambda. (h) Increases in both lambda and alpha produced gradual increases in the root-mean-square error (RMSE) between the filtered and their corresponding target spectra.

Envelope-shaped pseudospectra. Envelope-shaped pseudospectra derived from the synthetic test spectra are shown in Figure S2.

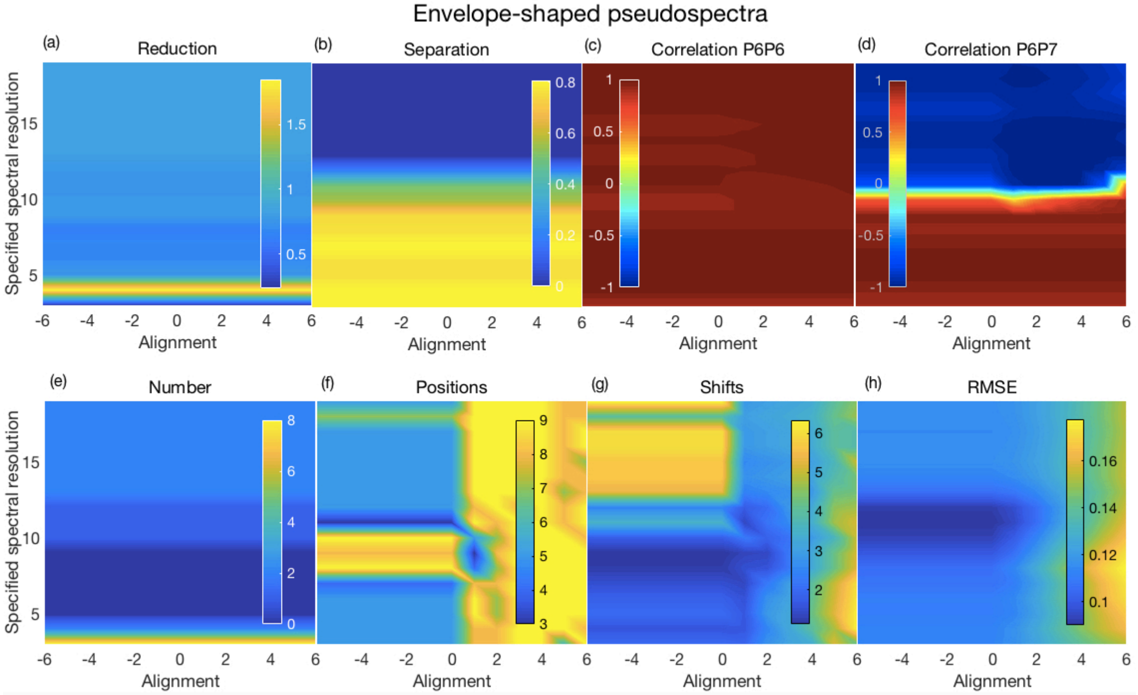

Figure S2. (a) Reduction in peak width with smaller numbers indicating larger reductions. Increases in the specified spectral resolution attenuated peak width reduction while changes in alignment of the pseudospectra with regard to the test spectra had no effect. (b) Separation between peaks with larger numbers showing more separation. Increases in the specified spectral resolution reduced the separation between peaks. (c) All correlations between P6 for both the target and pseudospectra were generally high. (d) In contrast, correlations between P6 and P7 of the pseudospectra changed rapidly from positive to negative near the specified spectral resolution corresponding to that of the truth set P1 (~12 channels). Alignment had minor effects. (e) The difference between the number of peaks counted in the pseudospectra and those present in the target spectra increased with increases in the specified spectral resolution. For specified spectral resolutions less than that of the truth set P1, the correct number of peaks tended to be present in the pseudospectra. Alignment had no effect. (f) The number of peaks in the pseudospectra with positions at variance with their corresponding positions in the target spectra exhibited complex behavior due to both the specified spectral resolution and the alignment. (g) Though also complex, the average extent of deviation in peak positions tended to increase with increasing specified spectral resolution and with some alignment values. (h) Increasing specified spectral resolution and alignment absolute values produced gradual increases in the RMSE between pseudospectra and their corresponding target spectra.

Blind deconvolution. Results of spectra blind deconvolved from the synthetic test spectra are shown in Figure S3A and from a blind deconvolution of those results in Figure S3B.

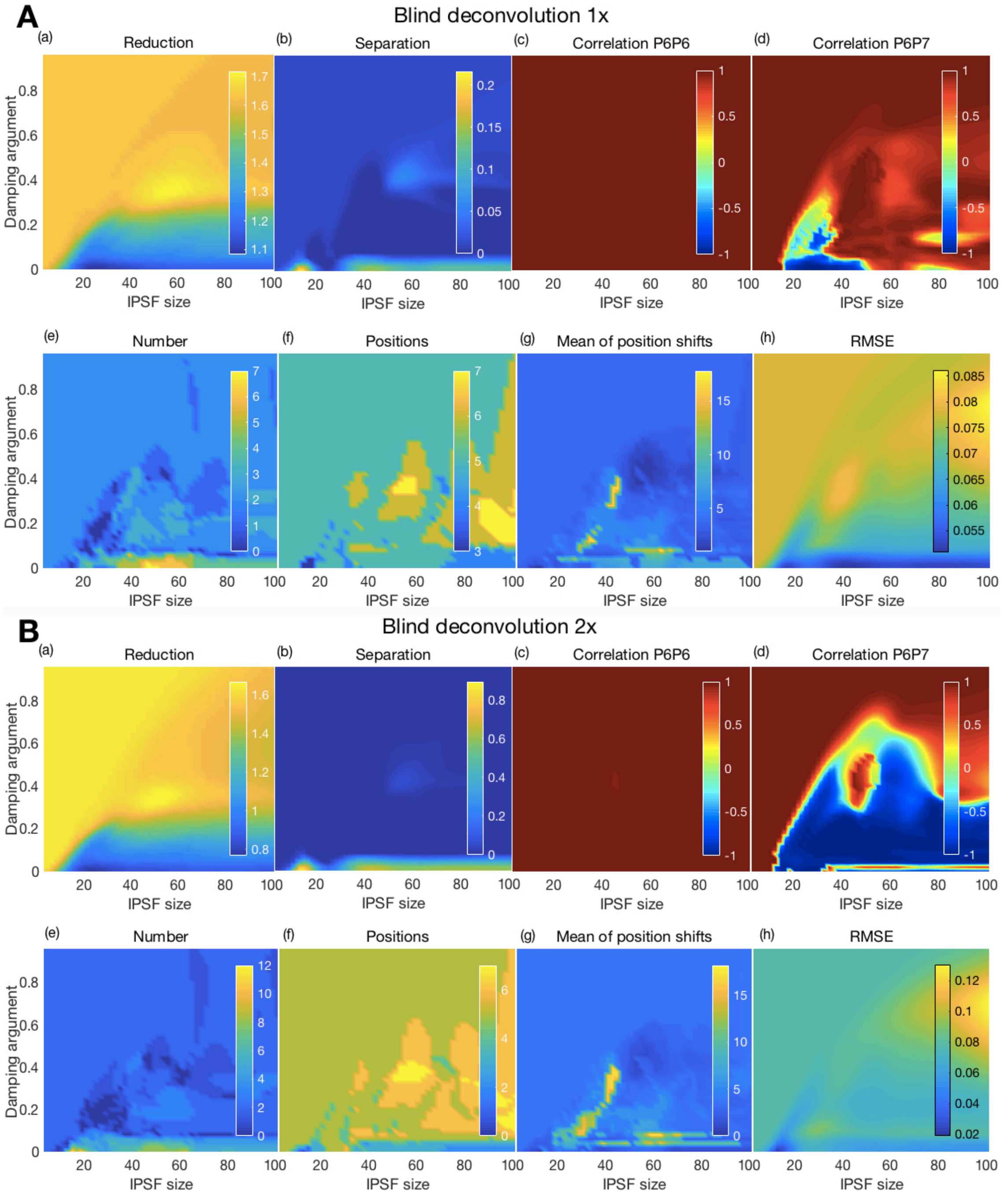

Figure S3. (A) results of blind deconvolution (blind deconvolution 1x) of the test spectra. (B) Blind deconvolution of the results in (A), blind deconvolution 2x, produced a general intensification of the effects in (A). (a) Reduction in peak width with smaller numbers indicating larger reductions. Increases in the damping argument increased peak widths while IPSF increases had little effect. (b) Separation between peaks with larger numbers showing more separation. Separation was mostly observed for small or zero values of the damping argument. The IPSF size had little effect. (c) Correlations between P6 for both the target and deconvolved spectra were

high everywhere. (d) In contrast, correlations between P6 and P7 of the deconvolved spectra showed complex behavior that changed to a more defined partition between positive and negative correlation with blind deconvolution 2x (in B). This suggested that blind deconvolution decorrelated these peaks for some values of the parameters. (e) The difference between the number of peaks counted in the deconvolved spectra and those present in the target spectra were low for moderate and high values of the damping argument but very high for low values and some IPSF sizes. This suggested the presence of small artefacts due to overdeconvolution that were not damped (see also the main text and Figure 4). However, (f) more non-artefactual peaks in the latter area tended to be correctly located and (g) while the others had smaller deviations from their correct positions. (h) The RMSE between doubly deconvolved spectra and their corresponding target spectra (in B) defined a small region of optimal performance with very small damping argument values and IPSF sizes between 15 and 20.

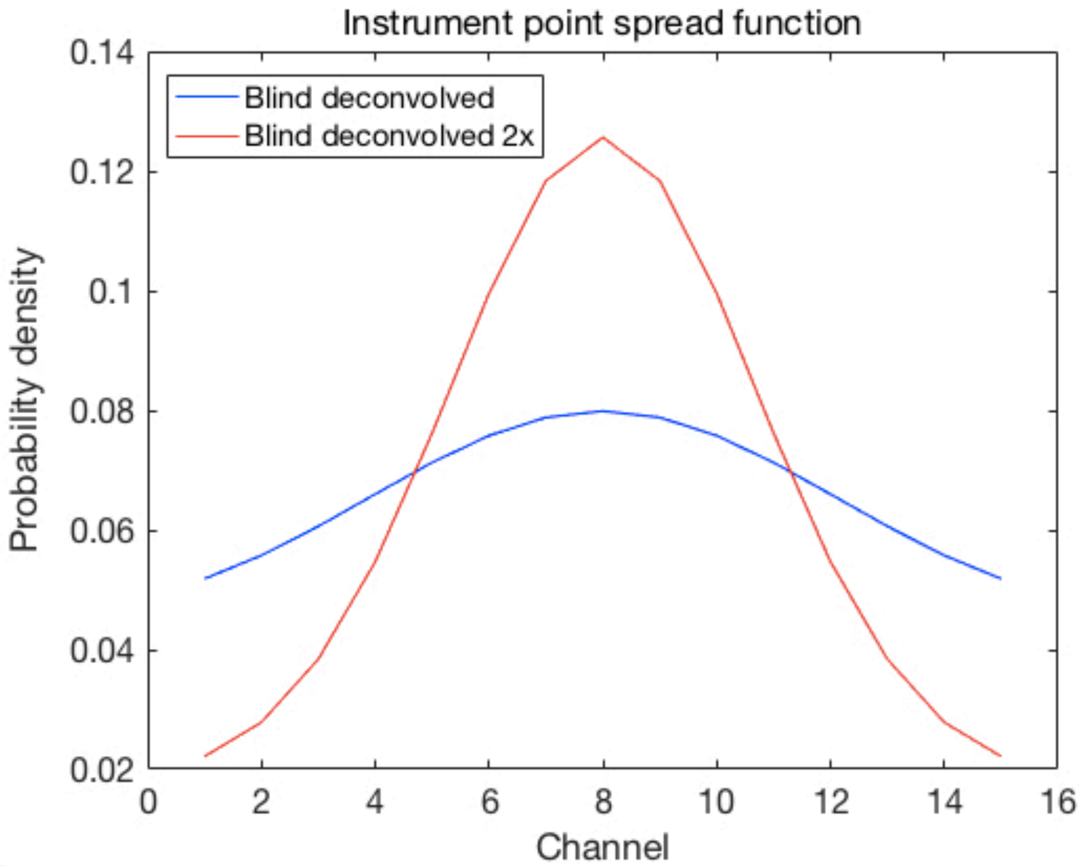

Figure S4. Estimated 15-channel IPSFs from the blind deconvolution of the test spectra (blue trace) and a successive blind deconvolution of the same spectra (red trace) show a narrowing of the IPSF consistent with the deconvolution of better resolved spectra.

Weighted overdeconvolution. The various figures of merit of resolved spectra obtained by overdeconvolution from test spectra are shown in Figure S5.

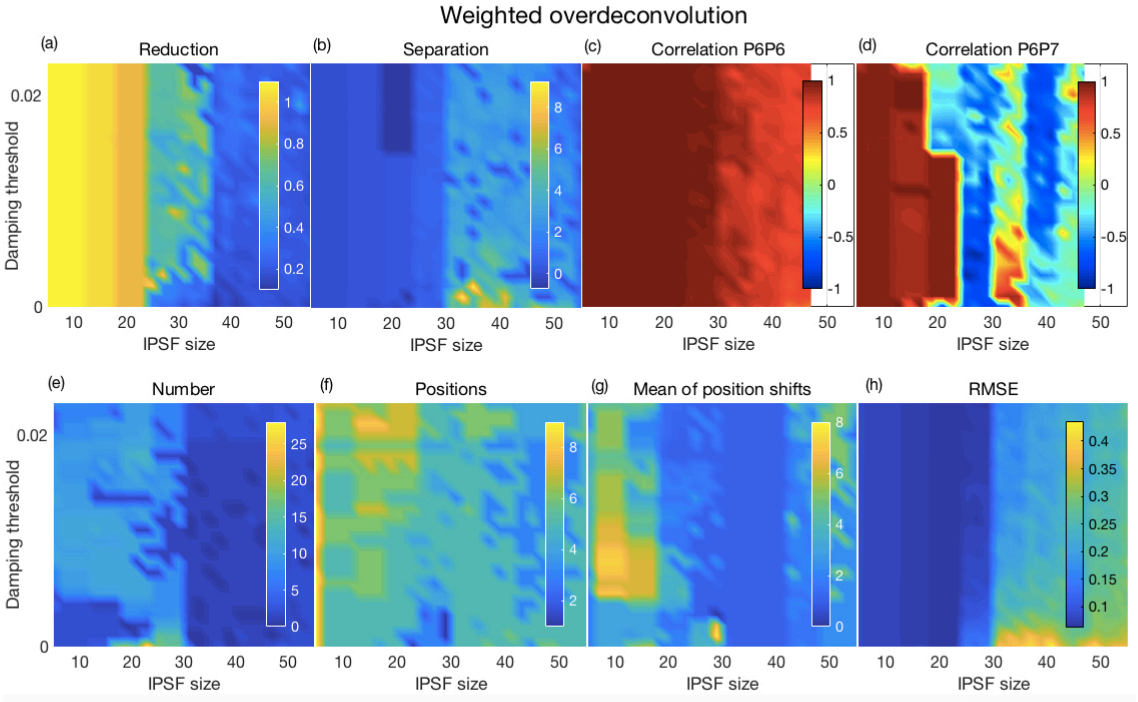

Figure S5. (a) Reduction in peak width with smaller numbers indicating larger reductions. Increases in the damping threshold had little effect while increases in IPSF size reduced peak widths. Likewise, (b) increased peak separation (larger numbers) was mostly dependent on increased IPSF sizes and less on the damping threshold. (c) Correlations between P6 for both the target and deconvolved spectra tended lower with increasing IPSF sizes. (d) In contrast, correlations between P6 and P7 of the deconvolved spectra decorrelated abruptly when IPSF sizes reached 20-25 channels. (e) The difference between the number of peaks counted in the deconvolved spectra and those present in the target spectra were low for moderate and large IPSF sizes but high for small IPSF sizes. (f) Both parameters affected the number of peaks with incorrect positions and (g) the deviations from their correct positions. (h) The RMSE between overdeconvolved spectra and their corresponding target spectra was mostly affected by IPSF size that showed a sharp transition near 30 channels.

Moving window peak fitting. The results of peak fitting to test spectra, obtained within a moving window, are shown in Figure S6. Peak widths were further reduced to 33% of the values obtained with the fits.

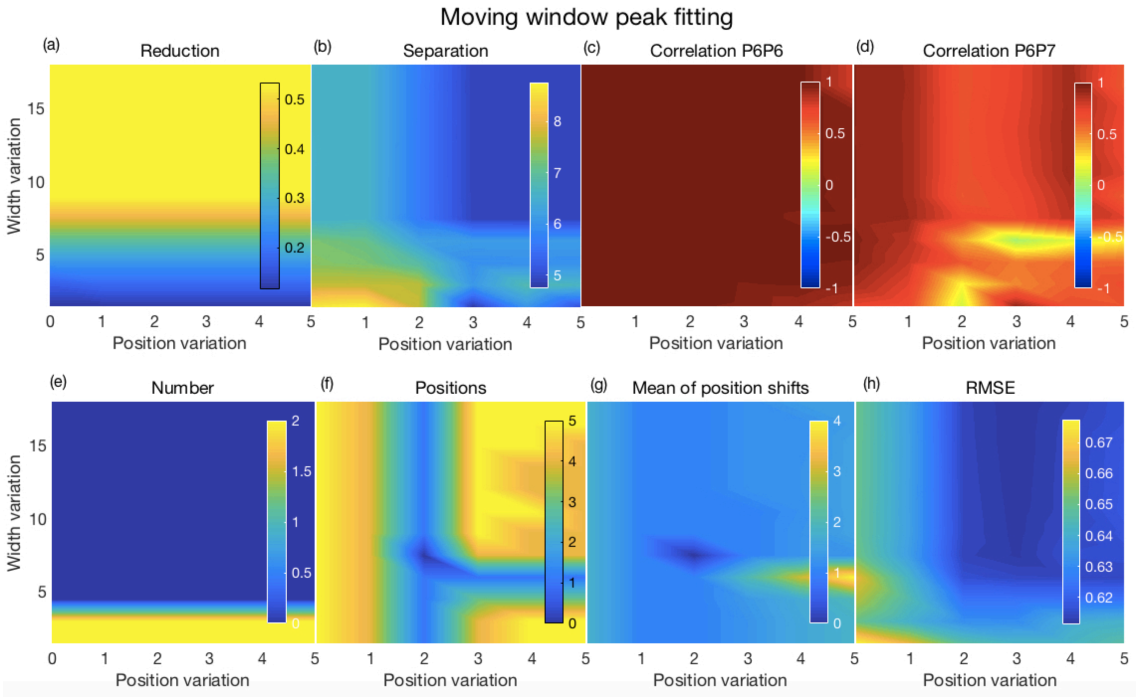

Figure S6. (a) Reduction in peak width with smaller numbers indicating larger reductions. Increases in the allowable width variation increased peak widths while increases in the allowable position variation had no effect. (b) Separation between peaks with larger numbers showing more separation. Both parameters contributed to changes in peak separation. (c) Correlations between P6 for both the target and deconvolved spectra were high everywhere. (d) Though generally high, correlations between P6 and P7 of the deconvolved spectra showed more complex behavior. This suggested that peak fitting decorrelated these peaks for only a few values of the parameters. (e) The difference between the number of peaks counted in the fitted spectra and those present in the target spectra increased when values of the allowable width variation were reduced below the HWHM of P1. Position variations had no effect. (f) The number of peaks with incorrect positions tended to be minimal for allowable position variations up to 2 channels while a similar effect was observed for allowable width variations up to near the P1 HWHM. The overlap of these minima produced an overall minimum for allowable peak and width variations up to 2 and up to 7.5, respectively, that (g) also occurred for the average deviations of peaks from their correct positions. (h) The RMSE between fitted spectra and their corresponding target spectra.

Peak fitting with *fityk*. The results of peak fitting to test spectra, obtained with the *fityk* program, are shown in Figure S7. Peak widths were further reduced to 33% of the values obtained with the fits.

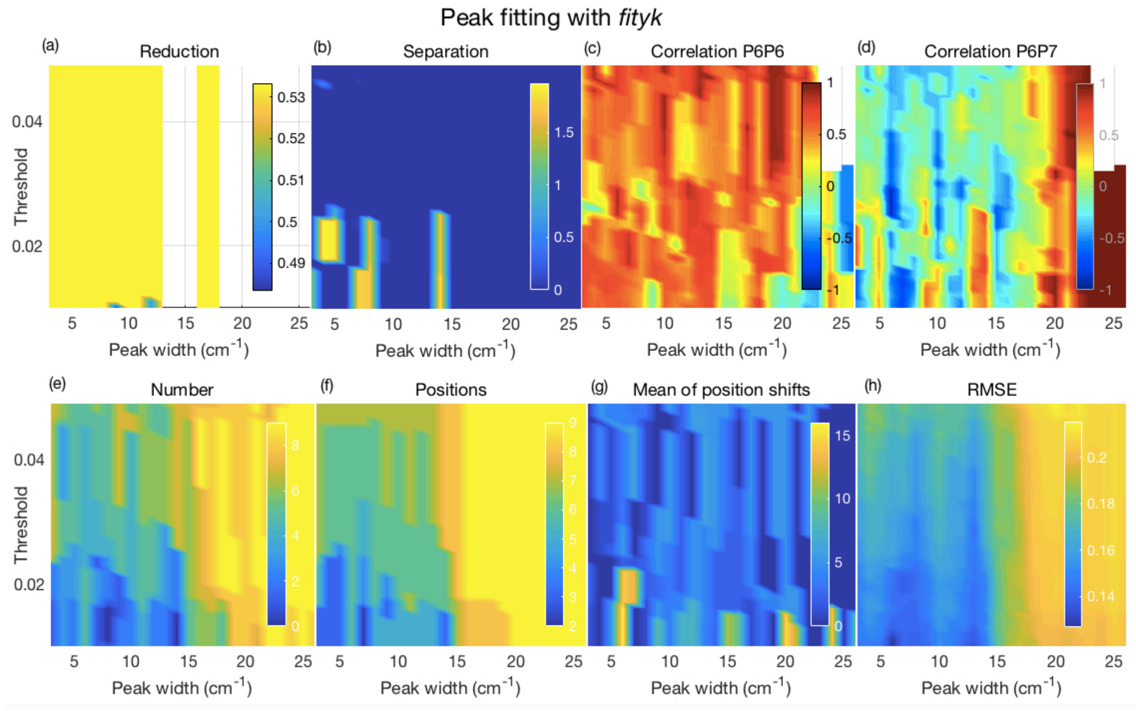

Figure S7. (a) Reduction in peak width with smaller numbers indicating larger reductions. As might be expected, threshold had no effect. Smaller specified initial peak widths yielded reduced fitted peak widths. Larger widths caused missed peaks. (b) Separation between peaks, with larger numbers showing more separation occurred in a few isolated regions where smaller amplitude thresholds and peak widths were specified. (c) Correlation coefficients between P6 for both the target and deconvolved spectra were generally modestly high, though with uneven variations. (d) Correlation coefficients between P6 and P7 of the deconvolved spectra were generally weak or somewhat negative, except being strongly positive for large peak widths. (e) A correct number of peaks were counted in the fitted spectra (that is, the same as the number present in the target spectra) in roughly the same regions where good peak separation occurred and (f) the number of peaks with correct positions also occurred in roughly the same regions. (g) The mean of shifts in peak positions seemed low overall – and artefact provoked by numerous small false peaks occurring near true ones. (h) The RMSE between fitted spectra and their corresponding target spectra was lower for smaller specified peak widths and lower peak amplitude thresholds.

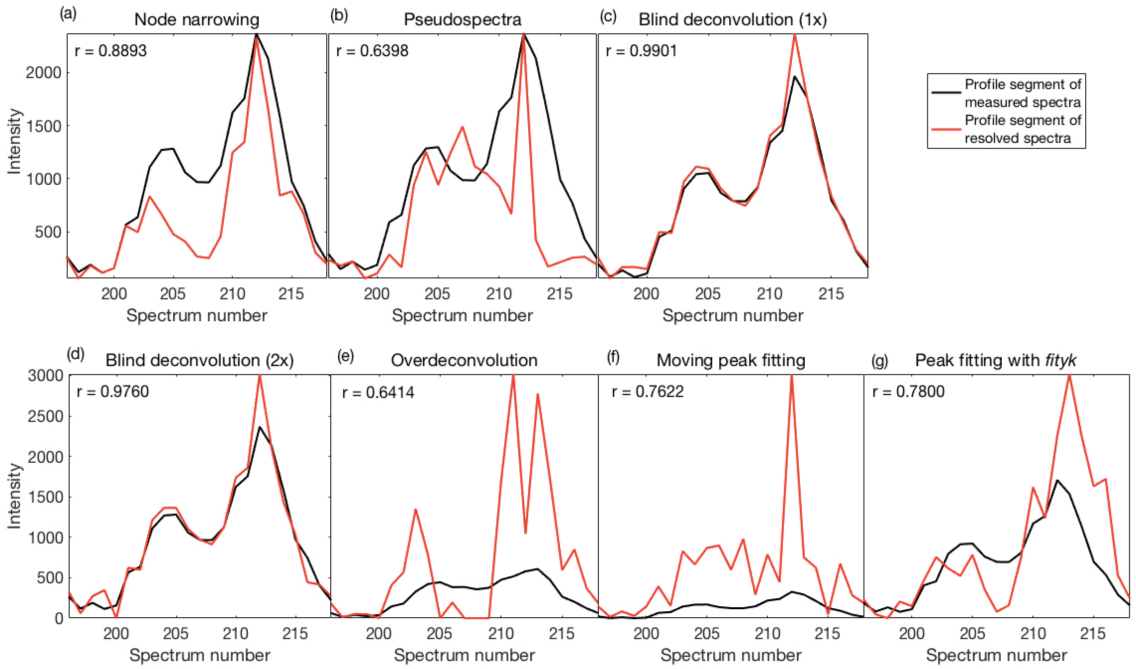

Figure S8. Example profile segments (spectrum 195 to spectrum 217 inclusive) of CHO cell spectra analyzed with 2DCOS and focused on the measured (black) and resolved (red) phosphatidylcholine peak near 717 cm^{-1}. Panels (a-g) show the results for the different methods as well as the correlation coefficients, r, for the measured and resolved segments. High correlation coefficients are not necessarily desirable because improved resolution might cause decorrelation of overlapping peaks, thus causing their true heights to differ from their apparent (i.e., unresolved) heights.

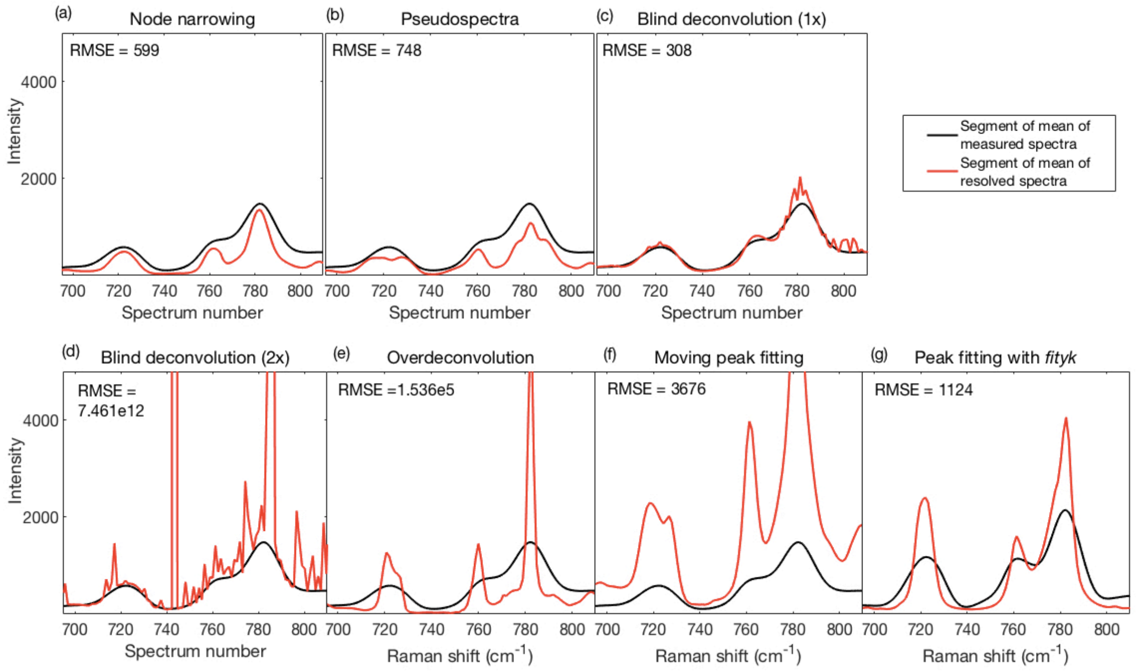

Figure S9. Panels (a-g) show the 695 to 810 cm^{-1} segment of the mean measured and mean resolved CHO cell spectra analyzed with 2DCOS. Also shown are the RMSE values between the mean measured and mean resolved spectra. All 462 spectra were included in the means, except for panel (g) where the means consisted of spectra 195 to 217 inclusive, as processing all 462 spectra proved difficult. Small RMSEs are not necessarily desirable as RMSE values will increase with improved resolution and higher RMSEs will occur with the deconvolution and fitting methods. However, RMSE values will also increase with aberrant results such as the large artefacts visible in the repeated blind deconvolution spectral segment in (d).
